# Sphingosine-1-phosphate receptor 3 regulates the transendothelial transport of high-density lipoproteins and low-density lipoproteins in opposite ways

**DOI:** 10.1093/cvr/cvad183

**Published:** 2023-12-18

**Authors:** Srividya Velagapudi, Dongdong Wang, Francesco Poti, Renata Feuerborn, Jerome Robert, Eveline Schlumpf, Mustafa Yalcinkaya, Grigorios Panteloglou, Anton Potapenko, Manuela Simoni, Lucia Rohrer, Jerzy-Roch Nofer, Arnold von Eckardstein

**Affiliations:** Institute of Clinical Chemistry, University of Zurich and University Hospital of Zurich, Rämistrasse 100, CH-8091 Zürich, Switzerland; Institute of Clinical Chemistry, University of Zurich and University Hospital of Zurich, Rämistrasse 100, CH-8091 Zürich, Switzerland; Department of Medicine and Surgery—Unit of Neurosciences, University of Parma, Parma, Italy; Department of Biomedical, Metabolic and Neural Sciences—Unit of Endocrinology, University of Modena and Reggio Emilia, Modena, Italy; Central Laboratory Facility, University Hospital of Münster, Münster, Germany; Institute of Clinical Chemistry, University of Zurich and University Hospital of Zurich, Rämistrasse 100, CH-8091 Zürich, Switzerland; Institute of Clinical Chemistry, University of Zurich and University Hospital of Zurich, Rämistrasse 100, CH-8091 Zürich, Switzerland; Institute of Clinical Chemistry, University of Zurich and University Hospital of Zurich, Rämistrasse 100, CH-8091 Zürich, Switzerland; Institute of Clinical Chemistry, University of Zurich and University Hospital of Zurich, Rämistrasse 100, CH-8091 Zürich, Switzerland; Institute of Clinical Chemistry, University of Zurich and University Hospital of Zurich, Rämistrasse 100, CH-8091 Zürich, Switzerland; Department of Biomedical, Metabolic and Neural Sciences—Unit of Endocrinology, University of Modena and Reggio Emilia, Modena, Italy; Institute of Clinical Chemistry, University of Zurich and University Hospital of Zurich, Rämistrasse 100, CH-8091 Zürich, Switzerland; Central Laboratory Facility, University Hospital of Münster, Münster, Germany; Institute of Laboratory Medicine, Marien-Hospital Osnabrück, Niels-Stensen-Kliniken, Osnabrück, Germany; Institute of Clinical Chemistry, University of Zurich and University Hospital of Zurich, Rämistrasse 100, CH-8091 Zürich, Switzerland

**Keywords:** Endothelium, Sphingosine-1-phosphate, HDL, LDL, SR-BI

## Abstract

**Aims:**

The entry of lipoproteins from blood into the arterial wall is a rate-limiting step in atherosclerosis. It is controversial whether this happens by filtration or regulated transendothelial transport.

Because sphingosine-1-phosphate (S1P) preserves the endothelial barrier, we investigated *in vivo* and *in vitro*, whether S1P and its cognate S1P-receptor 3 (S1P_3_) regulate the transendothelial transport of lipoproteins.

**Methods and results:**

Compared to apoE-haploinsufficient mice (CTRL), apoE-haploinsufficient mice with additional endothelium-specific knock-in of S1P_3_ (S1P_3_-iECKI) showed decreased transport of LDL and Evan’s Blue but increased transport of HDL from blood into the peritoneal cave. After 30 weeks of high-fat diet feeding, S1P_3_-iECKI mice had lower levels of non-HDL-cholesterol and less atherosclerosis than CTRL mice. *In vitro* stimulation with an S1P_3_ agonist increased the transport of ^125^I-HDL but decreased the transport of ^125^I-LDL through human aortic endothelial cells (HAECs). Conversely, inhibition or knock-down of S1P_3_ decreased the transport of ^125^I-HDL but increased the transport of ^125^I-LDL. Silencing of *SCARB1* encoding scavenger receptor B1 (SR-BI) abrogated the stimulation of ^125^I-HDL transport by the S1P_3_ agonist. The transendothelial transport of ^125^I-LDL was decreased by silencing of *SCARB1* or *ACVLR1* encoding activin-like kinase 1 but not by interference with LDLR. None of the three knock-downs prevented the stimulatory effect of S1P_3_ inhibition on transendothelial ^125^I-LDL transport.

**Conclusion:**

S1P_3_ regulates the transendothelial transport of HDL and LDL oppositely by SR-BI-dependent and SR-BI-independent mechanisms, respectively. This divergence supports a contention that lipoproteins pass the endothelial barrier by specifically regulated mechanisms rather than passive filtration.

## Graphical Abstract

**Graphical Abstract:**
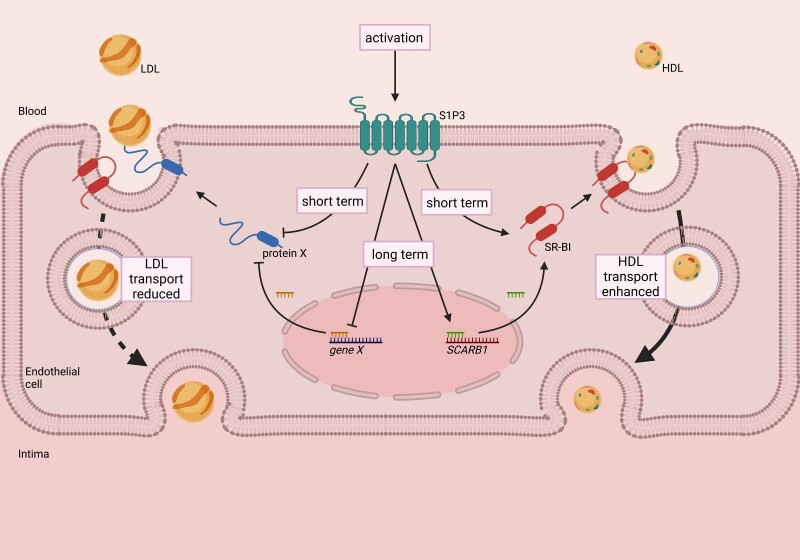

**Time of primary review: 23 days**

**See the editorial comment for this article ‘Can another lipid, sphingosine-1-phosphate, treat atherosclerosis?’, by W. Younis and I.J. Goldberg, https://doi.org/10.1093/cvr/cvae050.**

## 1. Introduction

According to the nowadays widely accepted ‘response-to-injury’ theory, the accumulation of low-density lipoproteins (LDL) in the arterial wall plays a pivotal role in the initiation and pathogenesis of atherosclerosis.^1,2^ Conversely, the removal of cholesterol from the intima by cholesterol efflux to high-density lipoproteins (HDL) and subsequent reverse cholesterol transport should confer protection against atherosclerosis.^3^ Two principal determinants define the accumulation of lipoproteins within the arterial wall: first, the concentration of lipoproteins in plasma which has been intensively investigated and successfully exploited to develop drugs which also prevent the incidence of cardiovascular events^1^; second, the entering and leaving of the arterial wall by LDL and HDL through mechanisms that have yet remained elusive.^2–4^ To reach the subendothelial space, both LDL and HDL must cross the intact endothelium. Traditionally, this transit is explained by passive filtration^4,5^ although as early as in the 1980s ultramicroscopic studies in rabbits indicated that aortic endothelial cells internalize and re-secrete LDL.^6^ The paradigm of passive filtration has also been challenged by the identification of several rate-limiting factors of transendothelial lipoprotein transport, namely scavenger receptor SR-BI, activin-like kinase 1 (ACVRL1), as well as caveolin-1 for LDL^7–10^ and SR-BI, ATP binding cassette transporter G1, endothelial lipase, and the ecto-ATPase/P2Y-receptor axis for HDL.^11–13^ Moreover, SR-BI was found to regulate the removal of HDL from extravascular tissues, including the arterial wall, into lymphatic vessels.^14,15^

Sphingosine-1-phosphate (S1P) is an endogenous lipid agonist of five G-protein coupled receptors termed S1P_1_, S1P_2_, S1P_3_, S1P_4_, and S1P_5_.^16^ In the endothelium, binding of S1P to the S1P_1_ or S1P_3_ receptors promotes the closure of intercellular junctions and hence the maintenance of the endothelial barrier.^17,18^ In parallel with reducing paracellular filtration of albumin, S1P was reported to increase the transcytosis of albumin through pulmonary microvascular endothelial cells.^19^ In this way S1P controls the trafficking of solutes, proteins, and cells between intra- and extravascular compartments.^18,20^ In mice, the knock-out of the S1P-binding protein apolipoprotein M (apoM) caused a strong decrease of S1P levels in plasma and HDL as well as increases in the permeability of lung capillaries and the blood–brain barrier for albumin suggesting the involvement of this compound in the transendothelial transport regulation.^19,21,22^ We previously demonstrated that apolipoprotein (apo)M and the S1P_1_ receptor promote rather than inhibit the transendothelial transport of HDL.^23^ We show here that the S1P_3_ receptor regulates the transendothelial transport of LDL and HDL in opposite directions both *in vitro* and *in vivo*.

## 2. Methods

### 2.1 Mouse models

Triple transgenic mice overexpressing murine S1P_3_ exclusively in endothelial cells were developed by crossing two lines. The *S1pr3^LoxP-STOP-LoxP (LSL)^* line, generated by genOway (Lyon, France) using their patented Rosa26 locus Quick knock-in^TM^ technology, carries a transgenic cassette in the Rosa26 locus, which harbours the *S1pr3* cDNA. It is separated from the synthetic cytomegalovirus early enhancer/chicken β-actin (*CAG*) strong promoter by a *LoxP-STOP-LoxP (LSL)* insert (see Supplementary material online, *Figure S1*). *S1pr3*^LSL^ mice were crossed to *Apoe*^−/−^*Cdh5-CreER^T2^* mice as described previously for *S1pr1*^LSL^ mice^23^ so that the *S1pr3* transcript is expressed exclusively in the endothelial cell lineage. We term these S1P_3_-inducible endothelial cell knock-in mice S1P_3_-iECKI mice. Double heterozygous *Apoe*^+/−^*Cdh5-CreER^T2^*^+/−^ mice were used as controls (CTRL). Genotyping was performed by classical PCR on DNA isolated from tail biopsies using the following primer sequences to identify the Cre-excised allele: For CATCAGGTTCTCCAAGACGATGAAGC; Rev AGCCTCTGCTAACCATGTTCATGCC (amplicon: 424 bp). The experiments on transport of lipoproteins and Evan’s Blue as well as en-face immunostaining of S1P_3_ and SR-BI were performed on 10–12 weeks old female mice fed a regular chow diet. For atherosclerosis studies, following induction of S1P_3_ overexpression, 6-week-old female mice received high-fat atherogenic diet (w/w: 1.25% cholesterol; 16% fat; 0,5% sodium cholate; Altromin, Lage, Germany; corresponding to Research Diets D12109) for 30 weeks. For euthanasia, animals were anaesthetized with 5% (v/v) isoflurane introduced via a vaporizer and then subjected to cervical dislocation. All experiments conformed to the guidelines from directive 2010/63/EU and were approved by the local animal protection authorities (LANUV, Recklinghausen, Germany, permit 84-02.04.2011.A351).

### 2.2 Immunostaining of aorta and quantification of atherosclerotic lesions

For immunostaining of S1P_3_ and SR-BI in the aortic endothelium as well as quantification of atherosclerosis we proceeded as described previously.^23^ For the immunostaining, we used the anti-S1P_3_ and anti-SR-BI antibodies from Novus Biologicals (NBP2-24762 and NB400-101, respectively) at concentrations of 20 μg/mL and secondary antibodies from Novus Biologicals (NBP1-76096 or NBP1-72973) at concentrations of 3.3 μg/mL.

### 2.3 Lipoprotein isolation and labelling

LDL (1.019 < *d* < 1.063 g/mL) and HDL (1.063 < *d* < 1.21 g/mL) were isolated from fresh human normolipidemic plasma by sequential ultracentrifugation as described previously.^24,25^ LDL and HDL were radio-iodinated with Na^125^I by the McFarlane monochloride procedure modified for lipoproteins.^25^ Specific activities between 300 and 900 cpm/ng of protein were obtained. For assessment of vascular permeability of lipoprotein in mice, LDL and HDL were labelled with DyLight^TM^ 550 fluorescent dye (DyL, Thermo Fischer, Schwerte, Germany) according to the manufacturer’s instruction and as described previously.^23^

### 2.4 Assessment of vascular permeability for lipoproteins and Evans Blue

As described previously,^23^ Evans Blue (600 µg/animal), DyL-LDL, or DyL-HDL (each 500 µg/animal) were injected in the tail vein of S1P_3_-iECKI, and CTRL mice 15 min prior to the *i.p.* injection of LPS (25 µg/animal). Mice were sacrificed after 3 h, and their peritoneal cavities were washed with 10 mL of ice-cold heparinized PBS. The cells were spun down, and the supernatants were analysed for Evans Blue with photometry (620 nm, FluoStar Optima, BMG LabTech, Ortenberg, Germany) and for DyL-LDL or DyL-HDL with fluorescence spectrometry (560 nm/590 nm, FluoStar Optima).

### 2.5 Cell culture

Human aortic endothelial cells (HAECs) from Cell Applications Inc. (304-05a) were cultured in endothelial cell basal medium (LONZA Clonetics CC-3156 or ATCC PCS-100-030) with 5% foetal bovine serum (GIBCO), 100 U/mL of penicillin and 100 µg/mL streptomycin (Sigma-Aldrich), supplemented with singleQuots (LONZA Clonetics CC-4176 or ATCC PCS-100-041, containing hFGF, hVEGF, hIGF-1, hEGF, hydrocortisone, ascorbic acid, heparin) at 37°C in a humidified 5% CO_2_, 95% air incubator.

### 2.6 Small interfering RNA transfection

Endothelial cells were reverse-transfected with small interfering RNA (Ambion silencer select, Life technologies) targeted to S1P_3_ (cat. no. s4455 and s4453), SR-BI (cat. no. s2648, s2649, and s2650) or LDLR (cat. nos. s224006, s224007, and s4) or ACVRL1 (cat. nos. s986, and s988) and non-silencing control (cat. no. 4390843) at a final concentration of 5 nmol/L using Lipofectamine RNAiMAX transfection reagent (Invitrogen, 13778150) in an antibiotic-free medium. All experiments were performed 72 h post-transfection, and the efficiency of transfection was confirmed with at least two siRNAs against each gene using quantitative RT-PCR and western blotting.

### 2.7 Quantitative real-time PCR

Total RNA was isolated using TRI reagent (Sigma T9424) according to the manufacturer’s instructions. Genomic DNA was removed by digestion using DNase (Roche) and RNase inhibitors (Ribolock, Thermo Scientific). Reverse transcription was performed using M-MLVRT (Invitrogen, 200 U/µL) following the standard protocol as described by the manufacturer. Quantitative PCR was done with Lightcycler FastStart DNA Master SYBR Green I (Roche) using gene specific primers as followed: *S1PR3* (For: TGA TCG GGA TGT GCT GGC; Rev: GAG TAG AGG GGC AGG ATG GTA), *SCARB1* (For: CTG TGG GTG AGA TCA TGT GG; Rev: GCC AGA AGT CAA CCT TGC TC), *LDLR* (For: AAGGACACAGCACACAACCA; Rev: CATTTCCTCTGCCAGCAACG), *ACVRL1* (For: CGA CTT CAA GAG CCG CAA TG; Rev: AGG ACT CAA AGC AGT CCG TG), normalized to *GAPDH* (For: CCC ATG TTC GTC ATG GGT GT; Rev: TGG TCA TGA GTC CTT CCA CGA TA).

### 2.8 Lipoprotein binding, cell association, and transport

The methods for the quantification of binding, association, and transport of radiolabelled HDL and LDL by endothelial cells have been previously described.^23^ Briefly, all assays were performed in DMEM (Sigma) containing 25 mmol/L HEPES and 0.2% bovine serum albumin (BSA) instead of fetal bovine serum (FBS). Where indicated, cells were pre-treated for 30 min at 37°C with either S1P_3_ agonist CYM-5541 (Cat No: SML0680, Sigma, 100 nM) or S1P_3_ inhibitor TY52156 (Cat No:2404, Axon Medchem, 110 nM). Following the pharmacological drug treatments, the cells were incubated with 10 µg/mL of ^125^I-HDL or ^125^I-LDL without (total) or with 40 times excess of non-labelled HDL/LDL (unspecific) for 1 h at 4°C for cellular binding and at 37°C for association experiments. Specific cellular binding and association were calculated by subtracting the values obtained in the presence of excess unlabelled HDL/LDL (unspecific) from those obtained in the absence of unlabelled HDL/LDL (total).

### 2.9 Inulin permeability

HAECs were cultured on trans-well inserts for 72 h, and cells were later treated with indicated pharmacological drug inhibitors as indicated for 30 min. Post-treatments, cells in the apical compartment were incubated with 2 mCi/mL of ^3^H-inulin, and the filtrated radioactivity was collected in the basolateral compartment after 1 h.^13^

### 2.10 Western blotting

Endothelial cells were lysed in RIPA buffer (10 mmol/L Tris pH 7.4, 150 mmol/L NaCl, 1% NP-40, 1% sodium deoxycholate, 0.1% SDS, with protease and phosphatase inhibitors (complete EDTA (Roche)). 30 μg of protein (quantified with the micro BCA protein assay kit Cat No: 23245 from Thermo Scientific) were separated on SDS–PAGE and trans-blotted onto PVDF membranes (GE Healthcare). Membranes were blocked in PBS-T supplemented with 5% milk or BSA and incubated either for 1 h at room temperature or overnight on a shaker at 4°C with primary antibodies against S1P3 (ab108370, Abcam), SR-BI (NB400-131, Novus), LDLR (ab52818, Abcam), or ALK1 (ab108207, Abcam), at a dilution of 1:1000 in the same blocking buffer. Membranes were incubated for 1 h at room temperature with HRP-conjugated secondary antibody (Dako) in blocking buffer at a dilution of 1:2500. Membranes were further incubated with chemiluminescence substrate for 1 min (Pierce ECL plus, Thermo Scientific) and imaged using Fusion Fx (Vilber). As the loading control TATA-binding protein (TBP) was immunodetected with primary antibody at 1:5000 (ab51841, Abcam) and secondary antibody at 1:10 000 dilutions.

### 2.11 Cell surface expression analysis of SR-BI and LDLR

Intact cells were biotinylated using 20 mg/mL EZ-Link sulpho-NHS-S-S-Biotin (Thermo Scientific) on ice with mild shaking. After 1 h, biotin was quenched with ice-cold 50 mM Tris pH 7.4. Cells were lysed in RIPA buffer (total cell lysate), and 200–500 µg of lysates were incubated with 20 µL of BSA-blocked streptavidin beads suspension (GE Healthcare) for 16 h at 4°C and pelleted by centrifugation; the pellet represents surface proteins. Proteins were dissociated from the pellet by boiling with SDS loading buffer containing 50 mM of DTT and analysed by SDS–PAGE and immunoblotted with SR-BI antibody (NB400-131, Novus), LDLR (ab52818, Abcam), and TBP (ab51841, Abcam).

### 2.12 FACS-based analysis of LDLR cell surface expression

Cell surface levels of LDLR were determined by flow cytometry; 72 h after transfection, the medium was removed, and the cells were washed twice with PBS and detached using Accutase (Sigma-Aldrich, A6964) for 5 min at 37°C. The cells were then collected using fluorescence-activated cell sorting (FACS) buffer (PBS containing 0.5% BSA and 0.05% NaN_3_), washed with ice-cold FACS buffer, and then incubated with Blocking Buffer (PBS containing 0.5% BSA and 2% FBS) for 1 h on ice. After blocking, the cells were incubated with the monoclonal anti-LDLR antibody, LDLR (Progen, cat. number 61087) diluted 1:25 in FACS buffer for 1 h on ice. After washing with FACS buffer, the cells were incubated with goat anti-mouse IgG (H + L) Cross-Adsorbed AlexaFluor 647-conjugated secondary antibody (Thermo Fischer Scientific cat. A-21236) diluted to a final concentration of 4 ug/mL in FACS buffer for 1 h on ice in the dark. Finally, prior to the acquisition, the cells were washed with FACS buffer and resuspended in ice-cold FACS buffer containing Propidium Iodide (PI) to a final concentration of 1 ug/mL. Cells incubated with the secondary antibody only were used as negative controls to determine the signal-to-noise ratio. Sample acquisition was carried out on a BD LSR II Fortessa (BD-Biosciences) and using BD FACSDIVA™ software. Data analysis was carried out using FlowJo version 10 (FlowJO LLC). Approximately 10^4^ events per condition recorded at the final gate (PI negative) were used for analysis.

### 2.13 Statistical analysis

The data sets for all validation experiments were analysed using the GraphPad Prism 5 software. The individual values (technical replicates) obtained in the control group from each experimental run were averaged, and the average was set to 100%. Further, the percentage difference of each individual technical replicate (of the control group and other treatment conditions) was calculated with respect to the average value of the control group (which was set to 100%). Comparison between the means of two or multiple groups was performed with two-tailed Student’s *t*-test and one-way analysis of variance for independent samples, respectively. Pairwise comparisons were performed thereafter with Student–Newman–Keuls post hoc test. The data were obtained from at least three independent experiments, performed in triplicates or quadruplets. Values are expressed as mean ± SEM. *P* < 0.05 was regarded as significant.

## 3. Results

### 3.1 Endothelial overexpression of S1P3 differentially regulates endothelial permeability for LDL, HDL, and Evans Blue in mice

**Figure 1:**
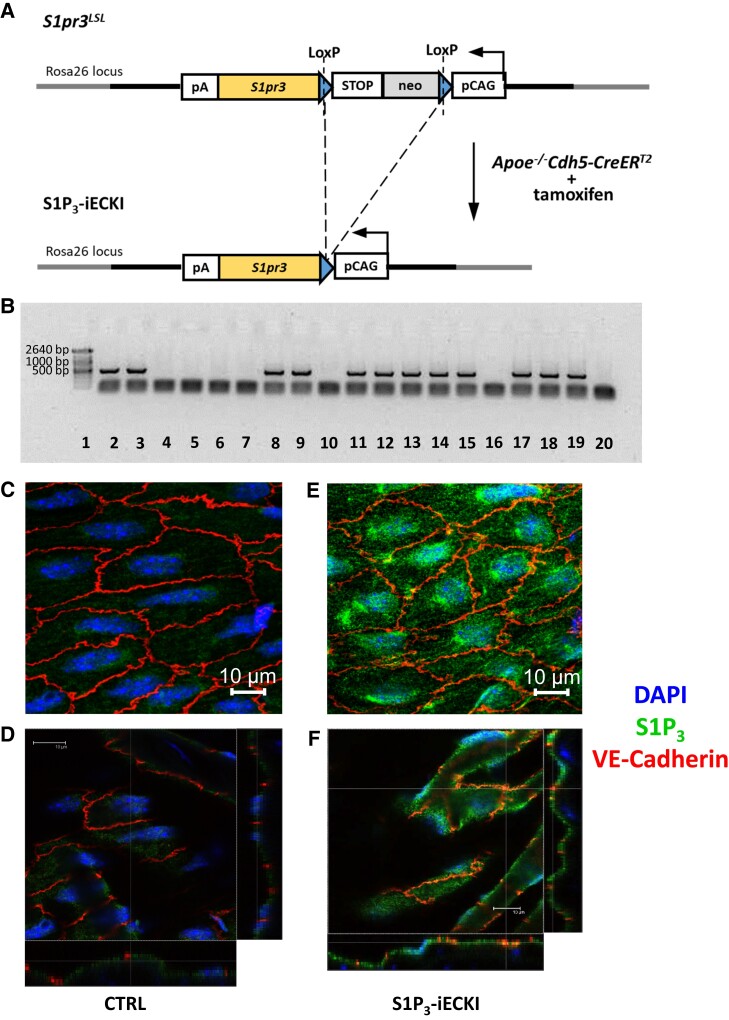
Targeting vector/transgene structure and Cre-mediated activation of transcription and demonstration of S1P_3_ in the endothelium of aortas from Apoe-haploinsufficient mice without (CTRL) or with overexpression of S1P_3_ (S1P_3_-iECKI). (*A*) The transgene, inserted within the ROSA26 locus via homologous recombination, contains the murine S1pr3 cDNA, separated from the synthetic cytomegalovirus early enhancer/chicken β-actin (CAG) strong promoter by a LoxP-STOP-Neomicin-LoxP (LSL) cassette. S1pr3LSL mice were crossed with the tamoxifen-inducible Apoe−/−Cdh5-CreERT2 mice, which express the Cre recombinase under the control of the VE-cadherin promoter, active in endothelial cells only. Gene overexpression was achieved by intraperitoneal injection of tamoxifen, and the LSL insert is hence excised only in Cre-expressing cells S1P_3_-iECKI: S1P_3_-inducible endothelial cell knock-in (mice). (*B*) Agarose gel electrophoresis of PCR-amplified genomic DNA from lungs of wild-type mice and S1P_3_-ECKI mice. From the left: lane 1, molecular weight marker (Marker XIV, Roche); lanes 2–19, 18 samples of mouse genomic DNA; lane 20, water. Upper bands (≍450 bp) in samples depicted in lanes 2, 3, 8, 9, 11, 12, 13, 14, 15, 17, 18, and 19 identify samples of S1P_3_-ECKI mice with the excision event. (*C–F*) En-face prepared aortas immunostainings. Aortas were quickly cleared from the adventitial tissue, opened longitudinally, and incubated with primary and secondary antibodies conjugated with green or red fluorescent dyes, as indicated. Nuclei were counterstained with 4'6-diamidino-2-phenylindole (DAPI). Images were captured by confocal microscope and *z*-axis projections of 14 scanned planes are shown. Scale bar = 10 μm. Original micrographs are shown as supplementary material online, *Figure S1*. (*D* and *F*) Orthogonal projections of single optical slices from *z*-stack. For each image, the right side and bottom side rectangles (the projections) clearly show the correspondence of the immunofluorescence signal with the endothelial lining. This view mode highlights also the localization of S1P_3_ receptors at the membrane level (red and green signals overlap generating orange and yellow signals).

To investigate the impact of the G-protein coupled receptor S1P_3_ on transendothelial lipoprotein transport *in vivo*, we generated *Apoe-*haploinsufficient mice which overexpress the human S1P_3_ receptor (S1P_3_-iECKI) under the control of a tamoxifen-inducible VE-cadherin promoter in endothelial cells only. Double heterozygous *Apoe*^+/−^*Cdh5-CreER^T2+^*^/−^ littermates were used as controls and comparators (hereafter termed CTRL) (*Figure 1A*). The successful knock-in and the increased expression of S1P_3_ were demonstrated by genotyping (*Figure 1B*) and immunofluorescence microscopy of aortas, respectively (*Figure 1C–F* and Supplementary material online, *Figure S1A* and *B*). Compared to CTRL mice (*Figure 1C* and *D*), the immunoreactivity for S1P_3_ was much enhanced in the aortas of S1P_3_-iECKI mice (*Figure 1E* and *F*). Upon feeding with a chow diet, plasma levels of cholesterol and triglycerides did not differ between S1P_3_-iECKI mice (1.29 ± 0.46 mmol/L and 0.78 ± 0.17, *N* = 6) and control mice (1.05 ± 0.19 mmol/L and 0.65 ± 0.07 mmol/L, *N* = 4). Upon gel filtration, no major difference in the distribution of cholesterol and triglycerides among lipoproteins was seen (not shown). After 30 weeks of feeding a 1.25% cholesterol-containing Western diet, the S1P_3_-iECKI mice had significantly lower levels of non-HDL-cholesterol and significantly higher levels of HDL-cholesterol as well as 60% less fatty lesions in their sinus aortae than Apoe-haploinsufficient CTRL mice (see Supplementary material online, *Figure S2*).

To assess the vascular permeability for albumin and lipoproteins in 10–12-week-old female S1P_3_-iECKI or CTRL mice fed a regular chow diet (5 or 6 per group) received intravenous injections of the albumin marker Evans Blue, DyL-LDL, or DyL-HDL 15 min prior to the *i.p* injection of LPS. S1P_3_-iECKI mice differed significantly from CTRL mice by 49% less uptake of DyL-LDL and 49% more uptake of DyL-HDL into the peritoneal cave (all *P* < 0.05). The 15% decrease in Evans Blue uptake was not statistically significant (*Table 1*). We also tested if the experiment can be performed in the absence of LPS, but we did not recover any fluorescence of DyL-LDL or DyL-HDL beyond background, if no LPS was injected i.p.(see Supplementary material online, *Table S1*).

**Table 1:** Endothelium-specific overexpression of S1P_3_ differentially regulates endothelial permeability for LDL, HDL, and Evan’s Blue in Apoe-haploinsufficient mice

Dye	CTRL	S1P_3_-KI
Evans’ Blue (arbU)	0.13 ± 0.01	0.11 ± 0.02
Fluorescent LDL (arbU)	89.0 ± 2.9	45.3 ± 4.7*
Fluorescent HDL (arbU)	18.5 ± 5.2	27.5 ± 2.9*

Intravenous (i.v.) injection of Evan’s Blue or DyLight-LDL or DyLight-HDL and i.p. stimulation with LPS. Collection of peritoneal fluid after 120 min.

*N* = 3–4 per group.

*P < 0.05, one-way ANOVA followed by Student–Newman–Keuls post hoc test.

### 3.2 S1P3 exerts an opposite effect on the transendothelial transport of HDL and LDL through human aortic endothelial cells

**Figure 2:**
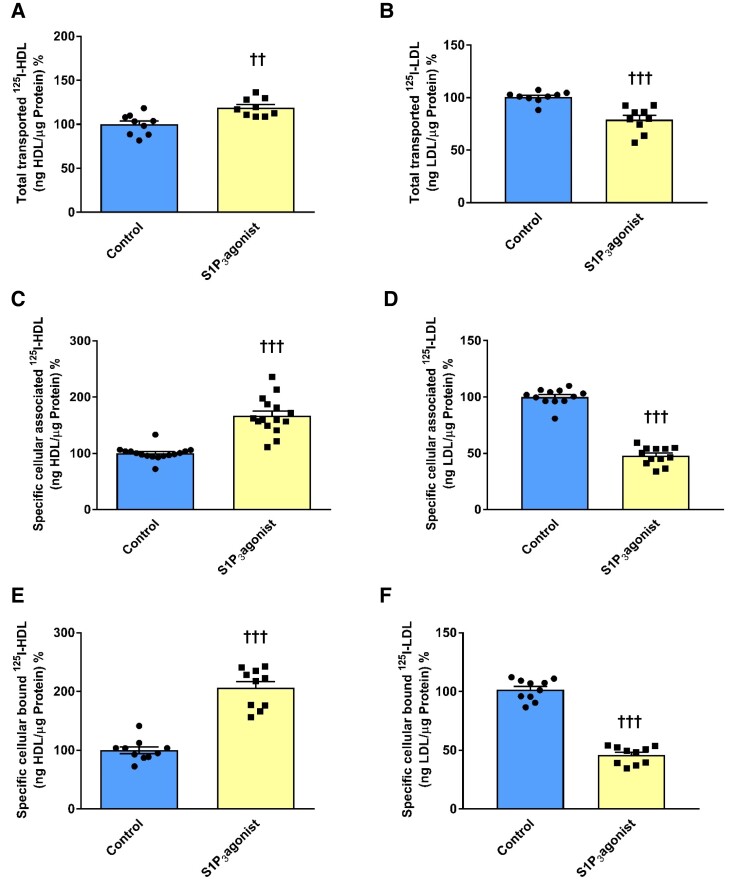
S1P_3_ agonist has opposite effects on transendothelial transport association and binding of HDL and LDL in HAECs. HAECs were cultured for 72 h. Cells were then treated with 100 nM CYM5541 for 30 min at 37°C. For the measurement of transport, HAECs were cultured on inserts. The transport of 10 µg/mL ^125^I-HDL (*A*) or 10 µg/mL ^125^I-LDL (*B*) from the apical to basolateral compartment was measured after 1 h incubation at 37°C. To study cellular binding and association, HAECs were incubated with 10 µg/mL of ^125^I-HDL or ^125^I-LDL for 1 h in the absence (total) or in the presence of 40-fold excess of unlabelled HDL and LDL, respectively, to record unspecific interactions. Specific binding and association were calculated by subtracting unspecific values from total values. To measure specific cell association, cells were incubated with ^125^I-HDL (*C*) or ^125^I-LDL (*D*) at 37°C The results are represented as means ± SEM of four independent triplicate experiments (*n* = 4). ^+++^*P* ≤ 0.001, ^++^*P* ≤ 0.01, ^+^*P* ≤ 0.05 (two-tailed Student’s *t*-test).

We next investigated whether S1P_3_ also regulates the transport of HDL and LDL through cultivated HAECs. First, we confirmed on both the mRNA and protein level the expression of S1P_3_ (see Supplementary material online, *Figure S3*). Previous RNA sequencing studies recorded mRNA of S1P_3_ in HAECs at levels that on average are lower than those of S1P_1_ but much higher than those of S1P_2_, S1P_4_, or S1P_5_ which were almost undetectable (see Supplementary material online, *Table S2*).^26–29^ Similar to the *in vivo* data, activation of S1P_3_ with the agonist CYM5541 at its IC50 concentration of 100 nM (see Supplementary material online, *Figure S4*) increased the transport of ^125^I-HDL (*Figure 2A*) but decreased the transport of ^125^I-LDL (*Figure 2B*). We then investigated whether these effects resulted from corresponding differences in the binding or uptake of the lipoproteins. Upon 30 min of treatment of HAEC with 100 nM of CYM5541 at 37°C, the specific cell association of HDL and LDL, which represents the combination of binding and uptake, was increased to 166% and reduced to 47%, respectively (*Figure 2C* and *D*). At 4°C, the specific cellular binding of ^125^I-HDL and ^125^I-LDL was increased to 206% and decreased to 45%, respectively (*Figure 2E* and *F*).

**Figure 3:**
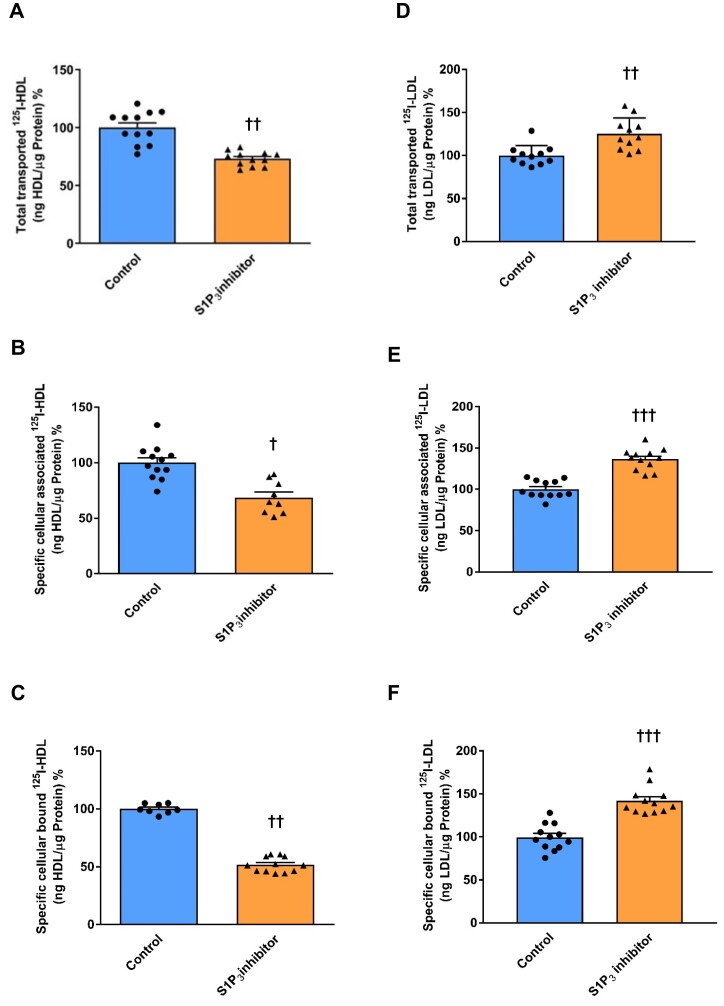
S1P_3_ inhibition has opposite effects on transendothelial transport, association, and binding of HDL and LDL in HAECs. HAECs were cultured for 72 h. Cells were then treated with 110 nM TY52156 for 30 min at 37°C. For the measurement of transport, HAECs were cultured on inserts. The transport of 10 µg/mL ^125^I-HDL (*A*) or 10 µg/mL ^125^I-LDL (*D*) from the apical to basolateral compartment was measured after 1 h incubation at 37°C. To study cellular association and binding, HAECs were incubated with 10 µg/mL of (*B* and *C*) ^125^I-HDL or (*E* and *F*) ^125^I-LDL for 1 h at 37°C and 4°C, respectively, in the absence (total) or in the presence of 40-fold excess of unlabelled HDL and LDL, respectively, to record unspecific interactions. (*B* and *E*) Specific association and (*C* and *F*) binding were calculated by subtracting unspecific values from total values. The results are represented as means ± SEM of four independent triplicate experiments (*n* = 4). ^+++^*P* ≤ 0.001, ^++^*P* ≤ 0.01, ^+^*P* ≤ 0.05, n.s. represents ‘not significant’ (two-tailed Student’s *t*-test).

**Figure 4:**
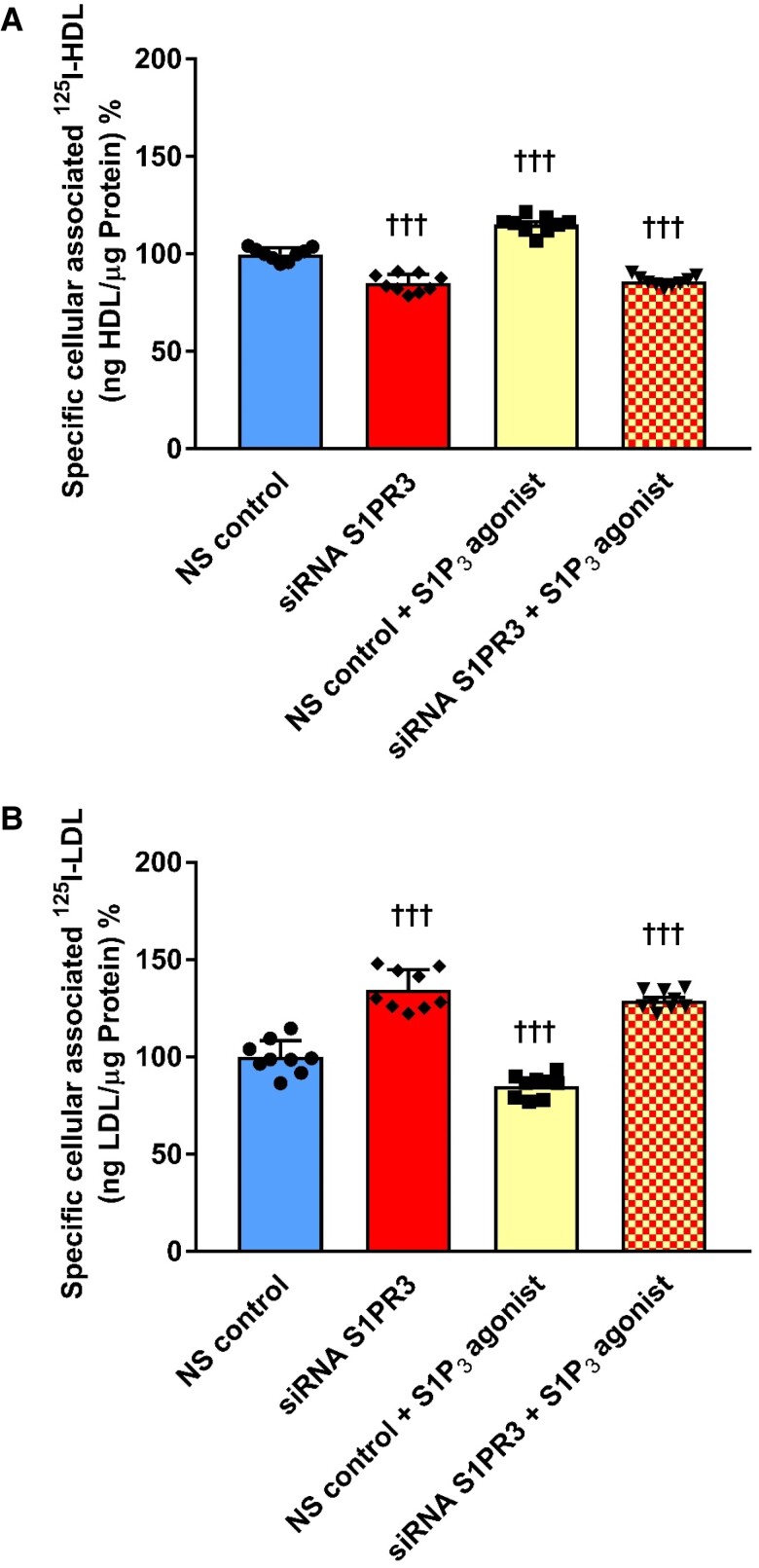
RNA interference with S1P_3_ counteracts the effects of CYM5541 on the association of HDL and LDL with HAECs. To study the specificity of S1P_3_ agonist treatment on the cellular association of ^125^I-HDL and ^125^I-LDL, HAECs were transfected with a specific siRNA against S1PR3 or with non-silencing control siRNA (NS control), and cellular association assays were performed 72 h post-transfection. Cells were then treated with 100 nM CYM5541 for 30 min, at 37°C. Cellular association of (*A*) ^125^I-HDL and (*B*) ^125^I-LDL were measured at 37°C by pre-treating cells with the S1P_3_ agonist. The results are represented as means ± SEM of three independent triplicate experiments (*n* = 3). ^+++^*P* ≤ 0.001 (Kruskal–Wallis one-way ANOVA followed by Student–Newman–Keuls post hoc test).

We then determined how pharmacological or RNA inhibition of S1P_3_ influences the endothelial interactions of radio-iodinated HDL and LDL. At the IC50 concentration of 110 nM (see Supplementary material online, *Figure S5*), the S1P_3_ inhibitor TY52156 did not interfere with the permeability of ^3^H-inulin (see Supplementary material online, *Figure S6*) through HAECs. TY52156 (110 nM) decreased the specific transport, association, and binding of ^125^I-HDL to 56% (*Figure 3A*), 68%, (*Figure 3B*), and 51%, respectively, of the untreated control (*Figure 3C*). Conversely, transport, association, and binding of ^125^I-LDL were increased to 137% (*Figure 3D*), 136% (*Figure 3E*), and 141%, respectively (*Figure 3F*). RNA interference strongly reduced S1P_3_ expression on both the mRNA (see Supplementary material online, *Figure S3A*) and protein levels (see Supplementary material online, *Figure S3B*) without affecting S1P_1_ expression (see Supplementary material online, *Figure S3A* and *C*) and decreased the cell association of ^125^I-HDL by about 20% (*Figure 4A*) but increased the cell association of ^125^I-LDL by about 40% (*Figure 4B*). Supporting the specificity of the S1P_3_ agonist, the stimulatory effect of CYM5541 on the association of ^125^I-HDL as well as its inhibitory effect on the association of ^125^I-LDL were abrogated by silencing of S1P_3_ (*Figure 4A* and *B*). Taken together, these results indicate that S1P_3_ regulates endothelial cellular binding, association, as well as transport of HDL and LDL in opposite directions.

### 3.3 S1P3 regulates cellular binding, association, and transendothelial transport of HDL via SR-BI

**Figure 5:**
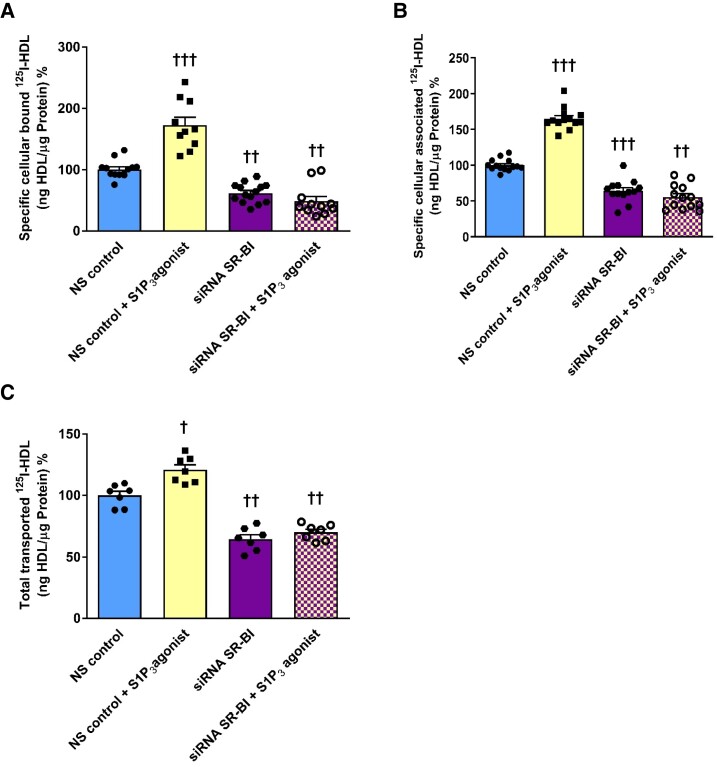
S1P_3_ agonists modulate SR-BI-dependent binding, association, and transport of HDL by HAECs. To study cellular binding, association, and transport of ^125^I-HDL, HAECs were transfected with a specific siRNA against SR-BI or with non-silencing control siRNA (NS control) and assays were performed 72 h post-transfection. Cells were then treated with 100 nM CYM5541 for 30 min, at 37°C. (*A*) cellular binding of ^125^I-HDL was measured at 4°C by pre-treating cells with the S1P_3_ agonist. (*B*) Cellular association of ^125^I-HDL was measured at 37°C by pre-treating cells with the S1P_3_ agonist. (*C*) The measurement of transport of ^125^I-HDL, and HAECs were cultured on inserts. The transport of ^125^I-HDL was measured by pre-treatment with the S1P_3_ agonist from the apical to basolateral compartment and was measured at 37°C. The results are represented as means ± SEM of four independent triplicate experiments (*n* = 4). ^+++^*P* ≤ 0.001, ^++^*P* ≤ 0.01, ^+^*P* ≤ 0.05 (Kruskal–Wallis one-way ANOVA followed by Student–Newman–Keuls post hoc test).

**Figure 6:**
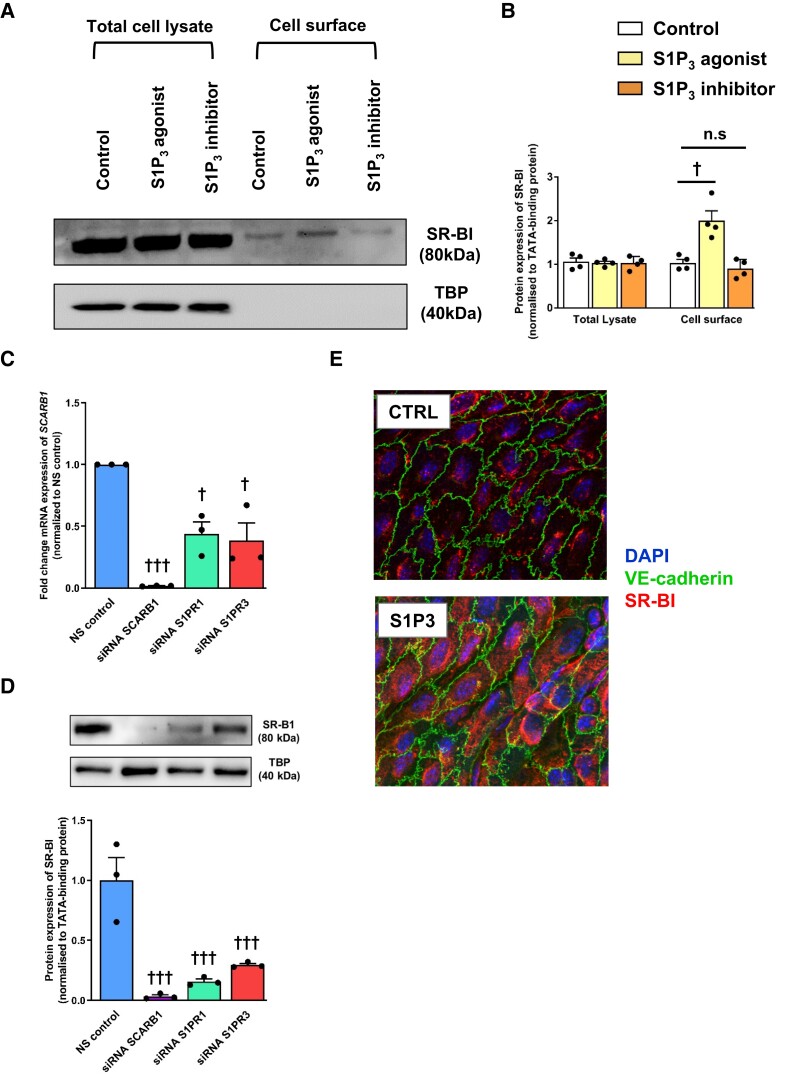
Effects of S1P_3_ on the expression of SR-BI. (*A* and *B*) Short-term pharmacological activation of S1P_3_ increases the cell surface expression of SR-BI. HAECs were cultured for 72 h. Cells were then treated with 100 nM CYM5541 or 110 nM TY52156 for 30 min at 37°C. (*A*) Cell surface expression of SR-BI in HAECs was measured using western blot analysis in total cell lysates (left) and on the cell surface (right). The western blots were probed with anti-SR-BI and anti-TBP (used as a control for intracellular protein expression). (*B*) The summary of four independent experiments quantified by densitometry. ^++^*P* ≤ 0.01 (two-tailed Student’s *t*-test). (*C* and *D*) Knock-downs of *S1P1* or *S1P3* dramatically decrease both SR-B1 protein and *SCARB1* mRNA expressions. HAECs were seeded at a density of 0.4 × 10^6^ cells/well in six-well plates. The cells were reverse-transfected with siRNA (10 nM) targeted to *SCARB1*, *S1P1*, or *S1P3*, or with non-silencing control siRNA (NC siRNA) for 72 h. The expression of SR-B1 protein and *SCARB1* mRNA was determined by western blot analyses and qRT-PCR, respectively. TATA-binding protein (TBP) and GAPDH mRNA were used as the internal control for western blot analyses and RT-qPCR, respectively. (*F*) Increased endothelial SR-BI expression in the aorta of S1P3-iECKI mice. Figures show en-face prepared aortic immunostainings of SR-BI in the endothelium of aortas from Apoe-haploinsufficient mice without (CTRL: upper part) or with overexpression of S1P3 (lower panel). Aortas were quickly cleaned of adventitial tissue, opened longitudinally, and incubated with primary and secondary antibodies conjugated with green or red fluorescent dyes, as indicated. Nuclei were counterstained with DAPI. Images were captured by confocal microscope, and *z*-axis projections of 14 scanned planes are shown. Scale bar = 10 μm. Original micrographs are shown as supplementary material online, *Figure S9*.

We previously reported that pharmacological activation of S1P_1_ increases the transendothelial transport of HDL by promoting the translocation of SR-BI to the cell surface. In addition, SR-BI immunoreactivity was increased in the aorta of S1P_1_ endothelial specific knock-in (S1P_1_-iECKI) mice.^23^ To determine whether S1P_3_ also regulates transendothelial transport of HDL through involvement of SR-BI, we combined pharmacological activation of S1P_3_ by CYM5541 with SR-BI silencing by siRNAs. The knock-down of SR-BI was efficient at the protein level (see Supplementary material online, *Figure S7A*). Silencing of SR-BI significantly decreased specific cellular binding, association, and transport by 40% (*Figure 5A*), 36% (*Figure 5B*), and 43% (*Figure 5C*), respectively. Activation of S1P_3_ failed to stimulate the cellular binding, association, or transport of ^125^I-HDL through endothelial cells when SR-BI was silenced (*Figure 5*). Cell surface biotinylation experiments showed that the activation of S1P_3_ receptor increases the cell surface abundance of SR-BI (*Figure 6A* and *B*; supplementary material online, *Figure S8*). The pharmacological inhibition of S1P_3_ with TY52156 did not decrease the abundance of SR-BI on the cell surface (*Figure 6A* and *B*; supplementary material online, *Figure S8*). However, RNA interference against S1P_3_ suppressed SR-BI expression both on the mRNA and on the protein levels (*Figure 6C* and *D*; Supplementary material online, *Figure S9*). RT-PCR of the distinct sequences revealed similar decreases of transcripts encoding SR-BI splice variants 1 and 2 (see Supplementary material online, *Figure S10*). Conversely, the knock-in of S1P_3_ increased the protein abundance of SR-BI in the aortic endothelium of S1P_3_-iECKi mice (*Figure 6E* and Supplementary material online, *Figure S11*). Thus, S1P_3_ appears to regulate SR-BI abundance by both fast post-translational and sustained transcriptional mechanisms.

### 3.4 S1P3 regulates transendothelial transport of LDL independently of LDLR, SR-BI, or ALK1

**Figure 7:**
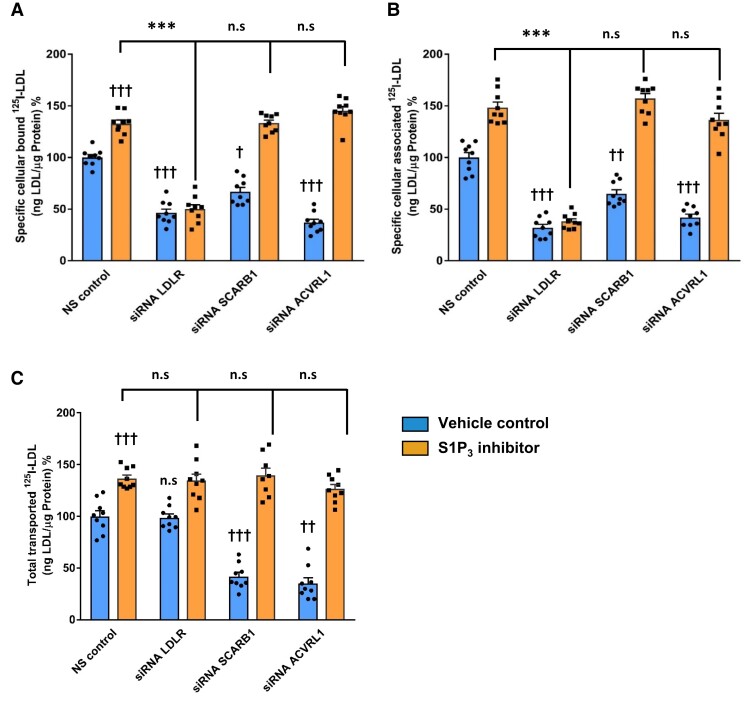
S1P_3_ regulates transendothelial transport of LDL independently of LDLR, SR-BI, and ACVRL1 in HAECs. HAECs were transfected with a specific siRNA against *LDLR* or *SCARB1* or *ACVRL1* or with non-silencing control siRNA (NS control) and assays were performed 72 h post-transfection. Cells were then treated with 110 nM TY52156 for 30 min at 37°C. (*A*) cellular binding of ^125^I-LDL was measured at 4°C by pre-treating cells with the S1P_3_ inhibitor. (*B*) Cellular association of ^125^I-LDL was measured at 37°C. (*C*) For the measurement of transport of ^125^I-LDL, HAECs were cultured on inserts. After pre-treatment with the S1P_3_ inhibitor, the transport of ^125^I-LDL from the apical to the basolateral compartment was measured at 37°C. The results are represented as means ± SEM of three independent triplicate experiments (*n* = 3). Vehicle control represents DMSO-treated condition. ^+++^*P* ≤ 0.001, ^++^*P* ≤ 0.01, ^+^*P* ≤ 0.05; ***P ≤ 0.001, **P ≤ 0.01, *P ≤ 0.05, n.s. represents ‘not significant’ (Kruskal–Wallis one-way ANOVA followed by Student–Newman–Keuls post hoc test).

To identify the targets for the increased binding, association, and transport of ^125^I-LDL upon S1P_3_ inhibition (*Figure 3D–F*), we tested the involvement of receptors known to promote the uptake of LDL into endothelial cells, namely LDLR, SR-BI, and ACVRL1^5,7,8^ by using RNA interference. Recorded at the protein level, the knock-downs of *SCARB1 (*coding for SR-BI), *LDLR*, and *ACVRL1* (coding for ALK1) were efficient but also caused some off-target effects. SR-BI was abolished upon knock-down of *SCARB1* but increased upon the knock-down of LDLR (see Supplementary material online, *Figure S7A*). LDLR protein was not detectable after the knock-down of *LDLR* but increased upon the knock-down of *SCARB1* or *ACVLR1* (see Supplementary material online, *Figure S7B*). ALK1 was abolished by knock-down of *ACVLR1* but also decreased upon silencing of *SCARB1* or *LDLR* (see Supplementary material online, *Figure S7C*). Silencing of *SCARB1 per se* decreased the cellular binding, association, and transport of ^125^I-LDL by 34%, 36%, and 59%, respectively (*Figure 7A–C*). Also, silencing of ACVRL1 *per se* decreased the cellular binding, association, and transport of ^125^I-LDL by 64, 59, and 65%, respectively (*Figure 7A–C*). However, the S1P_3_ inhibitor continued to stimulate the binding, association, and transport of ^125^I-LDL in the absence of either SR-BI or ALK1 (*Figure 7A–C*).

Silencing LDLR *per se* significantly decreased specific cellular binding and association of ^125^I-LDL by 54 and 68%, respectively (*Figure 7A* and *B*), but had no effect on the transport of ^125^I-LDL (*Figure 7C*). In the absence of LDLR, the S1P_3_ inhibitor failed to stimulate the cellular binding and association of ^125^I-LDL (*Figure 7A* and *B*). Flow cytometry analysis did not reveal any change in the cell surface expression of LDLR in response to drug treatment with either the S1P_3_ agonist or S1P_3_ inhibitor or after RNA interference with S1P_3_ (see Supplementary material online, *Figure S12*).

Taken together, these findings confirm that the transendothelial transport of LDL is limited by the abundance of SR-BI and ALK1 but not by LDLR. The data also indicate that S1P_3_ inhibition stimulates transendothelial transport of ^125^I-LDL independently of LDLR, SR-BI, or ALK1.

## 4. Discussion

It is controversial whether macromolecules including lipoproteins pass the endothelial barrier by active or passive transport. For a long time, the three-poremodel of Rippe and colleagues dominated the discussion.^30^ It was most extensively investigated by the analysis of protein transport from the blood stream into the peritoneal fluid but extrapolated to microcirculation in general. The peritoneum consists of a layer of mesothelial cells that is covered by a basement membrane and a thicker layer of connective tissue. The latter also contains capillaries and lymphatics. The endothelial cells of the capillaries rather than the mesothelium or connective tissue limit the transport of macromolecules including proteins from the bloodstream into the peritoneal cavity. The lymphatics are rather mediating the reverse transport out of the cavity back into the bloodstream via the thoracic duct.^31^ According to the three-pore model, small pores with a diameter of 0.6–1.0 nm allow the passage of small molecules only, whereas large pores with a diameter of 40–60 nm allow the transport of large macromolecules (i.e. also LDL with a Stokes diameter of 25 nm) without any restriction. Intermediate pores with a diameter of 8–12 nm would allow the transport of proteins with a Stokes diameter below or within this threshold, for example, albumin (6 nm) but also HDL (8–12 nm).^31^ Previous data on the regulation of endothelial barrier function by S1P also indicated rather unselective effects of S1P on transendothelial macromolecule transport by inducing the formation and maintenance of adherens and tight junctions^32^ and, hence, did not contradict the three-pore model. Decreases of bioactive S1P plasma levels lead to the exudation of albumin into the extravascular space and to lung oedema as well as increased permeability of the blood–brain barrier in apoM-knock-out animals.^19,21,22^ Conversely, increases of S1P plasma levels in mice lacking sphingosine kinase 2 were reported to reduce the permeability of peritoneal capillaries for dextran beads and LDL.^33^

In contrast to this classical model, our *in vivo* and *in vitro* experiments provide several pieces of evidence suggesting that the endothelial S1P-receptor S1P_3_ differentially regulates the transendothelial transport of pro-atherogenic LDL and potentially anti-atherogenic HDL: On the one hand and in line with the general notion of S1P as a stabilizer of endothelial barriers, the endothelial overexpression of human S1P_3_ in mice reduced the transendothelial transport of LDL and, less so, Evans Blue, a marker of albumin transport. Also, in accordance with an inhibitory role of S1P on transendothelial transport, the *in vitro* transendothelial transport by cultivated HAECs of LDL was decreased upon activation of S1P_3_ but increased upon inhibition of S1P_3_ by drugs or RNA interference with *S1P3*. On the other hand, and in contradiction to a general inhibitory effect of S1P on transendothelial macromolecule transport, the transendothelial transport of HDL was enhanced both *in vivo* and *in vitro* by the overexpression and activation, respectively, of S1P_3_, and diminished upon inhibition of S1P_3_. We previously reported similar opposing effects of S1P_1_ on the transendothelial transport of HDL and albumin both *in vivo* and *in vitro*.^23^ Like the S1P_3_-iECKI mice described in this paper, S1P_1_-iECKI mice with endothelium-specific knock-in of S1P_1_ showed decreased atherosclerosis as well as LDL and albumin transport but enhanced HDL transport into the peritoneum.^23^ Likewise in cultivated HAECs, the binding, association, and transendothelial transport of HDL were enhanced by the S1P_1_ agonist SEW2871 but decreased by the S1P_1_ inhibitor W146.^23^ Conversely, the S1P_1_ agonist and inhibitor suppressed and promoted, respectively, the binding, association, and transendothelial transport of LDL (see Supplementary material online, *Figure S13*). We postulate that the heterogeneous downstream responses account for the differential effects of S1P_3_ (and S1P_1_) on the transport of HDL and LDL through endothelial cells. In view of the principally identical effects of S1P_1_ and S1P_3_ on the transendothelial transport of HDL, on the one hand, and albumin and LDL on the other hand, it is interesting to note that the loss or gain of function of the one receptor is not compensated or neutralized by the other neither by counter-regulated expression nor by function. Likewise, S1P_1_ and S1P_3_ did not mutually compensate the adverse effects of their knock-downs on nitric oxide production and apoptosis inhibition.^31–33^ It was even suggested that the two receptors cooperate at least upon interaction with S1P delivered by apoM-containing HDL.^34^

Interference with SR-BI abrogated the enhanced binding, uptake, and transport of HDL elicited by the S1P_3_ agonist. S1P-receptor activation appears to promote transendothelial HDL transport by a similar mechanism as the activation of S1P_1_ by S1P^23^ or VEGF receptor 2 by VEGF-A,^35^ namely by increasing the cell surface abundance of SR-BI. This may be due to the fact that activation of receptor induces Akt phosphorylation,^36^ which we previously found to mediate the stimulatory effect of VEGF on both SR-BI translocation and binding, uptake, and transport of HDL by HAECs.^35^ In addition, sustained alterations of S1P_3_ expression (but also S1P_1_ expression),^23^ either *in vivo* by knock-in or *in vitro* by RNA interference, change the total protein expression of SR-BI. The parallel decrease of *SCARB1* mRNA and SR-BI protein in HAECs treated with siRNAs against either S1P_3_ or S1P_1_^23^ suggests that this happens on the transcriptional level. The stimulatory effects of the S1P_3_ (this paper) and S1P_1_ agonists^23^ on HDL transport were both prevented by RNA interference with *SCARB1*, suggesting a causal link between the increase in SR-BI cell surface expression, and increased HDL transport upon activation of these S1P receptors. However, the S1P_3_ inhibitor reduced HDL transport without reducing the cell surface abundance of SR-BI. Although we cannot rule out methodological limitations to assess any quantitative decrease in SR-BI cell surface abundance, one must also consider the possibility that S1P_3_ inhibition interferes with additional proteins interacting with HDL and limiting its uptake and transport. In fact, by applying chemoproteomic ligand-receptor capturing to the endothelial cell line EA.hy926, we recently found that HDL interacts with several cell surface proteins in the vicinity of SR-BI. Two of them are the TAM receptor family member MERTK and aminopeptidase N, whose knock-down also led to decreased HDL uptake into both EA-hy926 cells and HAECs.^37,38^

Confirming data from other labs as well as our own lab, we found that SR-BI also mediates the binding, association, and transport of LDL by HAECs.^8,10,39^ Therefore, it is surprising that the stimulation of S1P_3_ rather inhibited transendothelial LDL transport (*Figure 2D–F*). The S1P_1_ agonist SEW287 exerts the same effect (see Supplementary material online, *Figure S13*). By combining RNA interference with S1P_3_ inhibition, we confirmed SR-BI as a limiting factor for transendothelial LDL transport but ruled out that it contributes to the enhanced transendothelial LDL transport upon inhibition of S1P_3_ (*Figure 7*). Similarly, we previously showed in bovine aortic endothelial cells that VEGF promotes transendothelial transport of HDL in an SR-BI-dependent manner but has no effect on LDL transport.^35^ This discrepancy may be the result of different splice variants of SR-BI^39^ expressed by endothelial cells. The two most prominent ones differ by the 45 carboxyterminal amino acid residues. Only variant 1 of SR-BI contains the binding sites for the adapter proteins PDZK1 and DOCK4 which were shown to be essential for cell surface expression in hepatocytes and endothelial LDL-uptake, respectively.^10,40^ However, siRNA interference with S1P_3_ suppressed the two transcripts encoding SR-BI variants 1 and 2. It is therefore unlikely that differences in the regulation and function of the two splice variants of SR-BI explain the opposite effects of S1P_3_ on the transendothelial transport of HDL and LDL. It is also important to note that SR-BI is no endocytic receptor per se.^41^ Notably, in hepatocytes and steroidogenic cells, SR-BI binds both HDL and LDL, however without internalizing the entire particles. This suggests the contribution of endocytic co-receptors that are present in endothelial cells but neither in hepatocytes nor in steroidogenic cells. Our lab previously used chemoproteomic techniques to elucidate the surface proteome of endothelial cells and hepatocytes in general as well as specifically parts thereof that interact with HDL and anti-SR-BI antibodies. These analyses provided evidence of synapse-like many-to-many interactions between HDL and proteins of the cell surface. SR-BI is a core-protein in these interactions.^37,38^ It may hence be that S1P_3_ (and S1P_1_) activate not only SR-BI but several other members of this HDL synapse. Vice versa, S1P_3_ and S1P_1_ may limit other members of an LDL-synapse and thereby overrule the effects of SR-BI on the uptake of LDL into a transcellular transport itinerary. As an alternative explanation, one may hypothesize that S1P_3_ and S1P_1_ activate an inhibitor of transendothelial LDL transport. By combining RNA interference with S1P_3_ inhibition, we confirmed ACVRL1^7^ as a limiting factor for transendothelial LDL transport but ruled out that it contributes to the enhanced transendothelial LDL transport upon S1P_3_ inhibition. By RNA interference, we also showed the involvement of the LDL-receptor in cellular binding and association of LDL. However, and in agreement with previous reports,^7,42^ silencing of LDLR did not interfere with the transendothelial transport of LDL. LDL-uptake via the LDL-receptor leads to lysosomal degradation rather than re-secretion of LDL.^7,42^ Interestingly, interference with LDLR, prevented the stimulatory effect of S1P_3_ inhibition on binding and association of LDL by HAECs. However, in contrast with the regulatory effect of S1P_3_ on the cell surface expression of SR-BI, neither the activation, the inhibition nor the knock-down of S1P_3_ altered the cell surface abundance of LDLR in HAECs (not shown). It thus appears that S1P_3_ regulates endocytic LDLR functions. This may be an important regulatory step in the removal of S1P from the plasma as LDLR was suggested to bind apoM-containing LDL and thereby contribute to S1P clearance.^43^ However, neither LDLR nor ALK1 explains the opposite effects of S1P_3_ and S1P_1_ on the transendothelial transport of HDL and LDL.

In conclusion, we here showed that S1P_3_ regulates the endothelial binding, uptake, and transport of HDL and LDL in an antagonistic manner. By inhibiting the transendothelial transport of pro-atherogenic LDL and promoting the transendothelial transport of potentially anti-atherogenic HDL, S1P_3_ may play an important role in the pathogenesis of atherosclerosis and serve as an interesting target for protection against atherosclerosis, following the example of the anti-ALK1 antibody which in a preclinical model reduced atherosclerosis by interfering with transendothelial LDL transport.^44^ In fact, several animal studies showed that genetic or pharmacological interference with S1P generation and degradation by sphingosine kinase 2^45,46^ and S1P lyase,^47^ respectively, the S1P-binding protein apoM,^23,48^ or S1P receptors^49–52^ affect the development of atherosclerosis. Most but not all studies support the anti-atherogenic role of S1P. In line with this, we found atherosclerosis reduced in mice with the endothelium-specific overexpression of S1P_1_^24^ or S1P_3_ (this study). However, it is important to keep in mind that even in the endothelium S1P and its receptors elicit several potentially anti-atherogenic effects beyond the regulation of transendothelial lipoprotein transport,^53^ for example, on the transmigration of leucocytes and nitric oxide production.^54^ Therefore, and because of the cholesterol-lowering effects of both the S1P_1_ and the S1P_3_ knock-ins, we cannot conclude any causal link between reduced atherosclerosis and beneficially altered transendothelial lipoprotein transport. Further studies are needed to show the pathogenic relevance of the differential regulation of transendothelial LDL and HDL transports through S1P and its cognate receptors S1P_1_ and S1P_3_.

## Supplementary Material

cvad183_Supplementary_Data

## Data Availability

The data underlying this article are available in the article and in its online supplementary material.

## Notes

Translational perspective The entry of lipoproteins from blood into the arterial wall is a rate-limiting step in atherosclerosis. It is controversial whether this happens by filtration or regulated transendothelial transport. The antagonistic effects of activated or inhibited sphingosine-1-phosphate receptor S1P3 on HDL and LDL through endothelial cells support the hypothesis that transendothelial lipoprotein transport occurs by specific mechanisms and pathways. By inhibiting the transendothelial transport of pro-atherogenic LDL and promoting the transendothelial transport of potentially anti-atherogenic HDL, S1P 3 (but also S1P1) may play an important role in the pathogenesis of atherosclerosis and serve as an interesting target for protection against atherosclerosis.
