# Supplementary material for: Sphingosine-1-phosphate receptor 3 regulates the transendothelial transport of high-density lipoproteins and low-density lipoproteins in opposite ways
Source: Cardiovasc Res. 2023 Dec 18;120(5):476–89. doi: 10.1093/cvr/cvad183 (PMC11060483; doi:10.1093/cvr/cvad183)
Supplement: cvad183_Supplementary_Data [file cvad183_supplementary_data.pdf]

Supplementary Tables and Figures

Sphingosine-1-phosphate receptor 3 regulates the transendothelial transport of HDL and LDL in opposite ways

Srividya Velagapudi, PhD[1,], Dongdong Wang, PhD[1*], Francesco Poti, PhD[2,3,*], Renata Feuerborn PhD[4], Jerome Robert, PhD[1], Eveline Schlumpf M.Sc[1], Mustafa Yalcinkaya, PhD[1], Grigorios Panteloglou, PhD[1], Anton Potapenko, PhD[1], Manuela Simoni, MD, PhD[3], Lucia Rohrer, PhD[1], Jerzy-Roch Nofer, MD MBA [4,5 *], and Arnold von Eckardstein, MD[1,*, $]*

1. Institute of Clinical Chemistry, University of Zurich and University Hospital of Zurich, Switzerland
2. Department of Medicine and Surgery - Unit of Neurosciences, University of Parma, Parma, Italy
3. Department of Biomedical, Metabolic and Neural Sciences - Unit of Endocrinology, University of Modena and Reggio Emilia, Modena, Italy
4. Central Laboratory Facility, University Hospital of Münster, Germany
5. Institute of Laboratory Medicine, Marien-Hospital Osnabrück, Niels-Stensen-Kliniken, Osnabrück, Germany

*: equal contributions

§ Present address: Molecular Cardiology, University of Zürich, Wagistrasse 12, CH 8952 Schlieren, Switzerland

$: Corresponding author: Arnold von Eckardstein, MD, Institute of Clinical Chemistry, University Hospital of Zürich, Switzerland, Rämistrasse 100, CH-8091 Zürich, Switzerland

Supplementary Table 1: Effect of lipopolysaccharide (LPS) on the recovery of fluorescence in the peritoneal fluid of wild type mice

	**Without LPS**	**With LPS**
PBS control	19.5 +/- 0.7	
DyL-LDL in the peritoneal fluid (arbU)	18.3 +/- 5.1	74.0 +/- 2.8
DyL-HDL in the peritoneal fluid (arbU)	22.3 +/-11.0	78.0 +/- 11.3

i.v. injection of PBS, DyLight-LDL or DyLight-HDL (350µg/animal) with or without i. p. stimulation with lipopolysaccharide (25.0 µg/animal). N = 3 per condition

S1PR1	S1PR2	S1PR3	S1PR4	S1PR5	platform	Assession number
19.41 (11.62)	0.12 (0.02)	2.41 (0.023)	1.14 (0.85)	0 (0)	Illumina HiSeq 4000 (Homo sapiens)	GSE89970
13.33 (1.53)	100.67 (24.11)	36.67 (9.29)	0.67 (1.15)	19.33 (4.51)	Illumina NovaSeq 6000 (Homo sapiens)	GSE199709
3044.67 (327.87)	29.04 (1.77)	3690.84 (444.962)	12.67 (5.13)	3 (1.73)	Illumina HiSeq 2500 (Homo sapiens)	GSE186524
91.0 (1.31)	0.39 (0.05)	2.07 (0.03)	0.25 (0.01)	0 (0)	Illumina NovaSeq 6000 (Homo sapiens)	GSE204771
4119 (1113.96)	23 (5.20)	751.55 (136.67)	3.67 (0.58)	0 (0)	Illumina NovaSeq 6000 (Homo sapiens)	GSE202119

Supplementary table 2: Expression of S1P receptors S1PR1, S1PR2, S1PR3, S1PR4 , and S1PR5 in human aortic endothelial cells (HAECs) as assessed by RNAsequencing. Numbers present means and standard deviations (in brackets)

Supplementary Figure 1

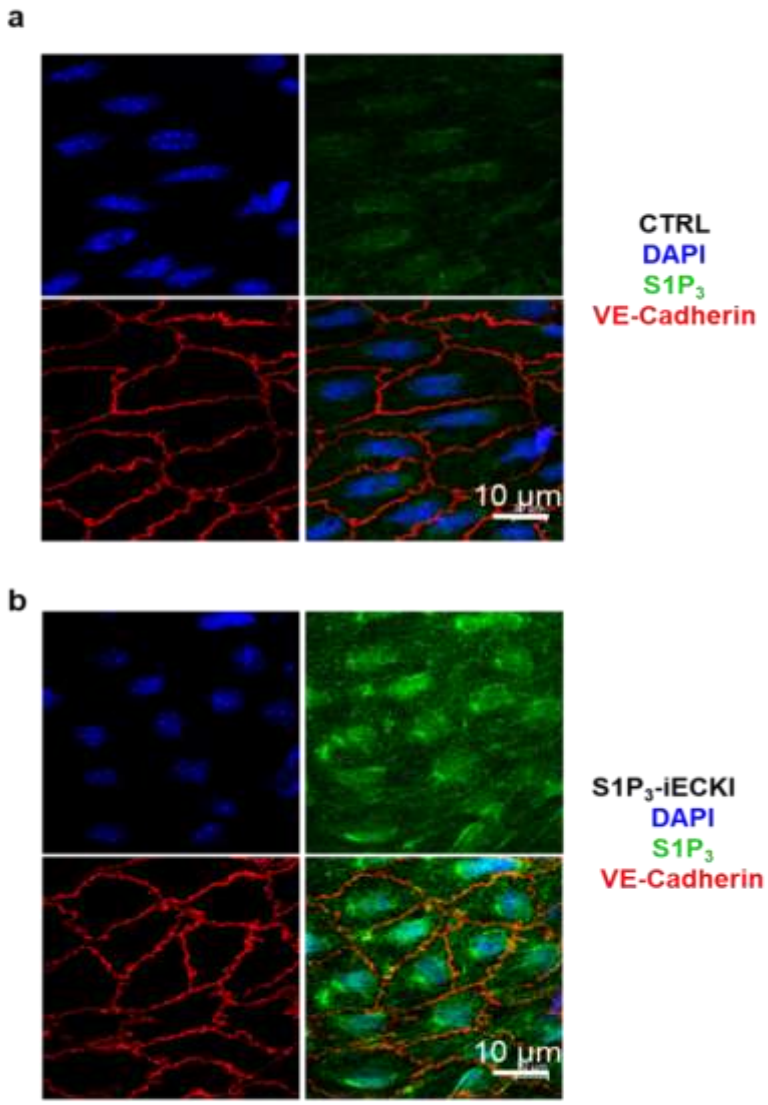

Supplementary Figure 1: Demonstration of S1P3 in the endothelium of aortas from *Apoe* haploinsufficient mice without (CTRL) or with overexpression of S1P3 (S1P3-iECKI). Figure shows *en-face* prepared aortas immunostainings. Aortas were quickly cleared from the adventitial tissue, opened longitudinally, and incubated with primary and secondary antibodies conjugated with green or red fluorescent dyes, as indicated. Nuclei were counterstained with DAPI. Images were captured by confocal microscope and z-axis projections of 14 scanned planes are shown. Scale bar = 10µm.

Supplementary Figure 2

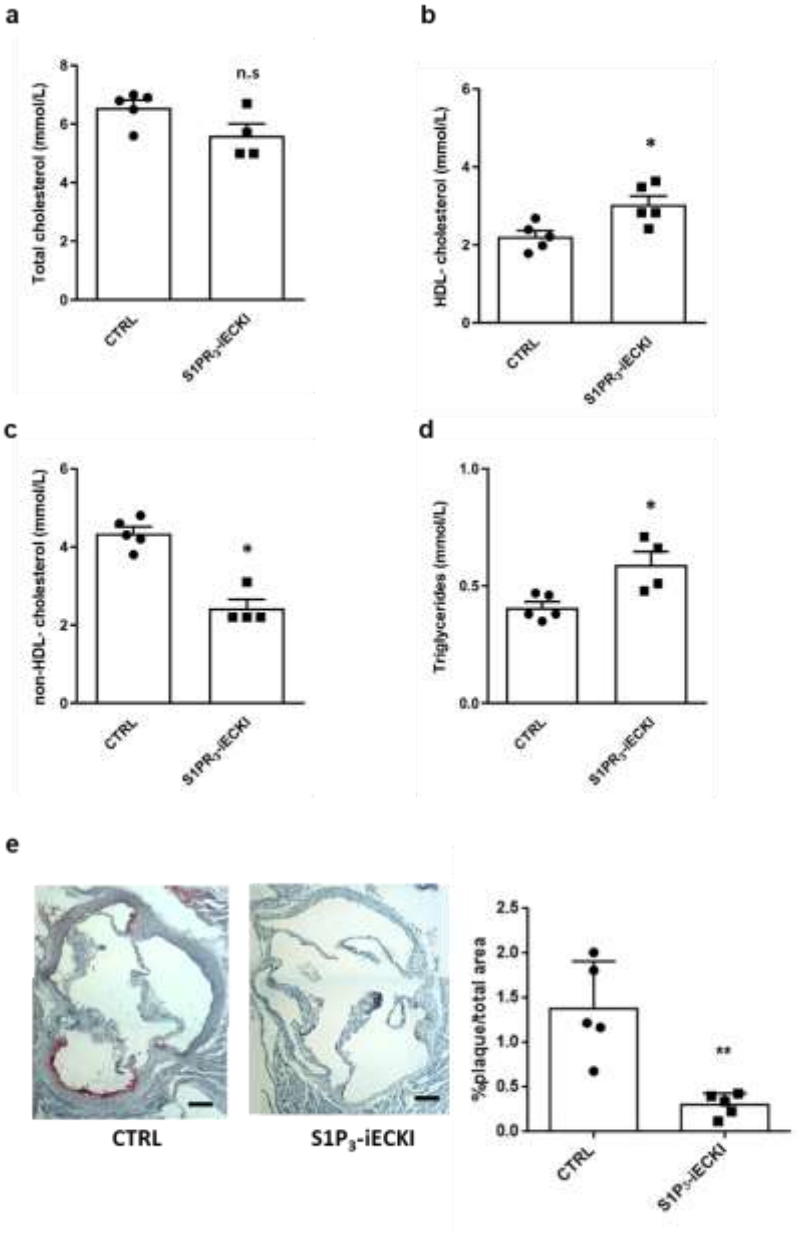

Supplementary Figure 2: Less severe hypercholesterolemia and fatty streak formation in S1P3-iECKI mice fed with a high fat diet *Apoe* haploinsufficient mice with (S1P3-iECKI) or without (CTRL) a knock-in of *S1P3* were fed for 30 weeks with a western diet containing 1.25% cholesterol before they were sacrificed for collection of blood and aortas. (Results are presented as means ± SEM; *: P calculated by Mann-Whitney U-Test).

Supplementary Figure 3

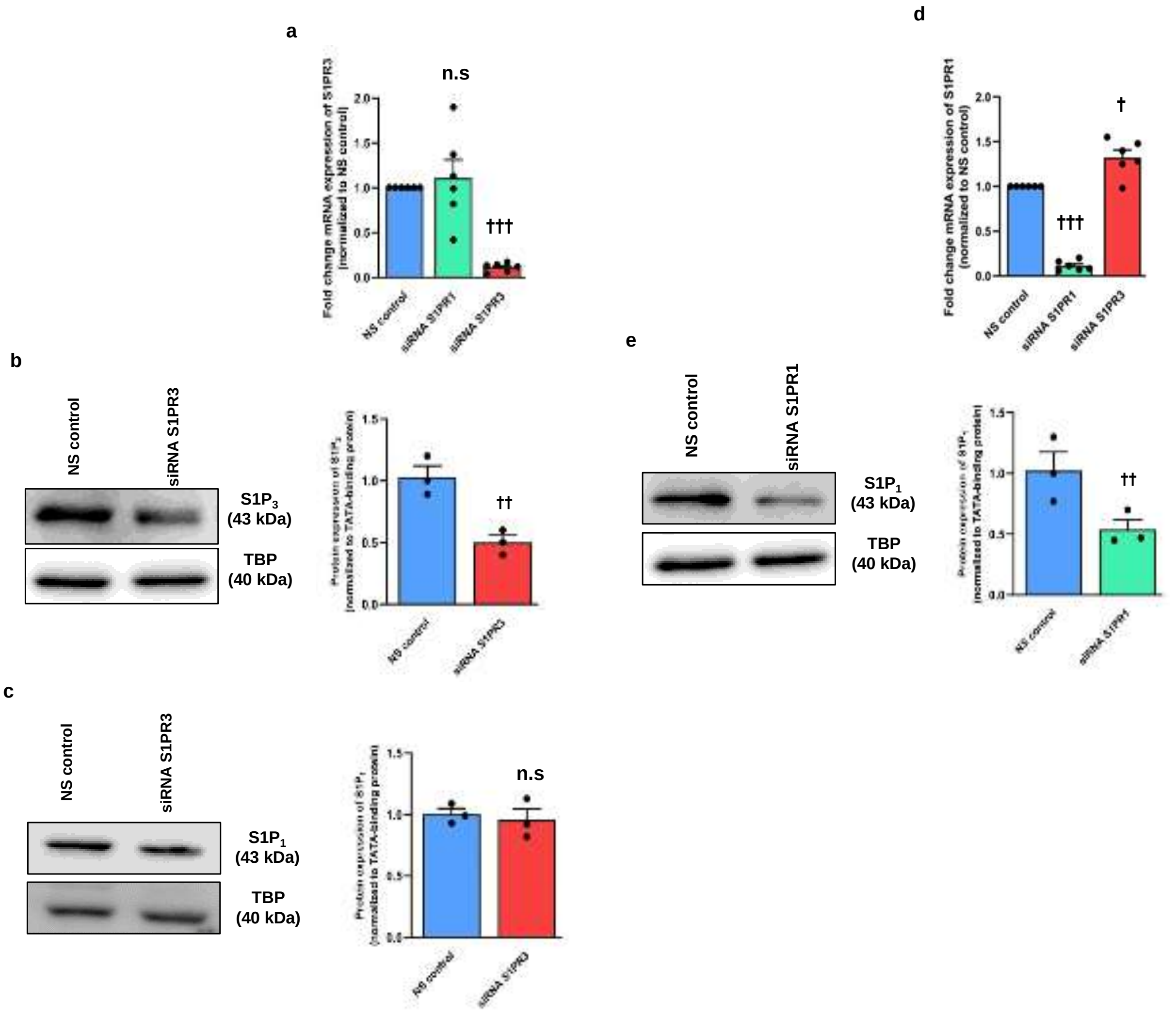

Supplementary Figure 3: Expression and Knock-down efficiency of $S1P_1$ and $S1P_3$ HAECs were transfected either with siRNA against *S1PR1 or S1PR3* or with non-silencing siRNA (NS control) for 72hours. mRNA expression of *S1PR3 (a)* and *S1PR1 (d)* normalized to GAPDH, Representative western blots and quantification (n=2-3) of $S1P_3$ (**b, c**) and $S1P_1$ **(e)** relative to TATA-binding protein (TBP) used as the loading control.

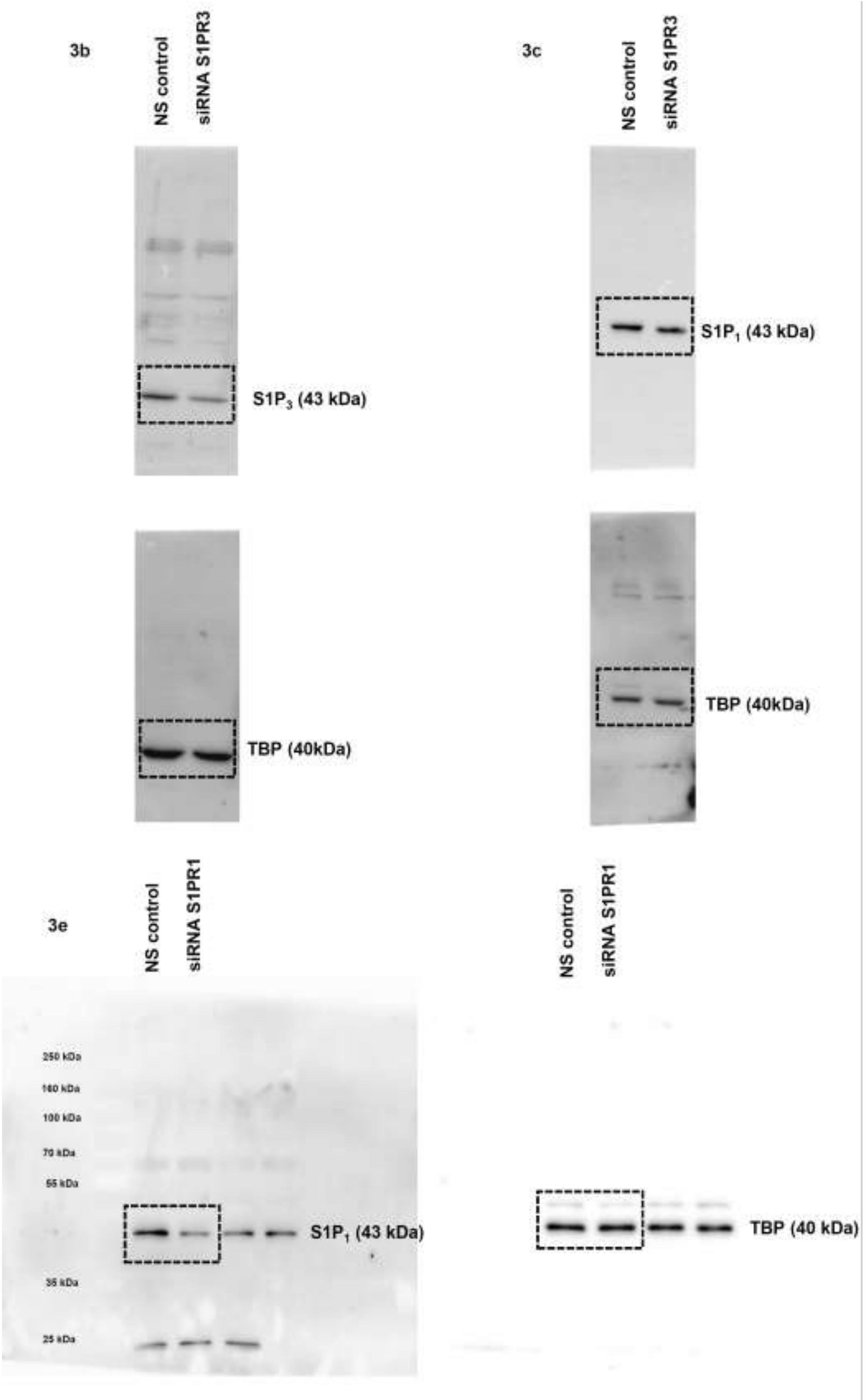

Supplementary Figure 3, continued: Complete and unedited gels of supplementary figures 3b, 3c and 3d.

Supplementary Figure 4

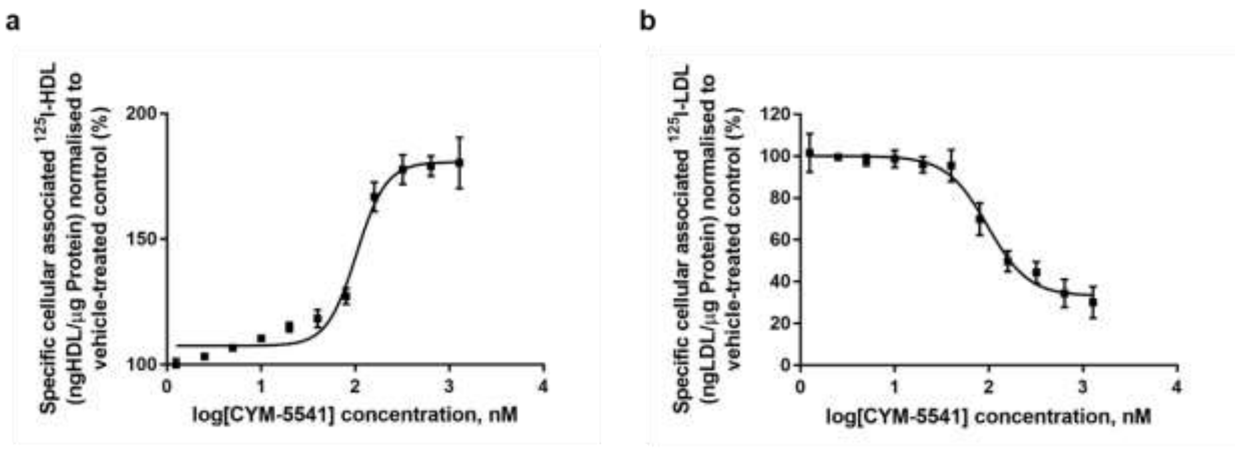

Log EC50 = 2.014 [103nM]
95% CI Log EC50 = 1.921-2.1 [83nM-126nM]

Log EC50 = 1.987 [95nM]
95% CI Log EC50 = 1.896-2.097 [79nM-125nM]

Supplementary Figure 4: Dose-dependent effects of the $S1P_3$ agonist CYM5541 on the specific association of ^{125}I-HDL (a) or ^{125}I-LDL (b) with human aortic endothelial cells (HAEC). HAEC were cultured for 72hours and treated for 30 minutes with increasing concentrations of CYM5541. HAECs were then incubated at 37 ºC for 1hour with 10µg/mL of ^{125}I-HDL (**a**) or ^{125}I-LDL (**b**) in the absence (total) or in the presence of 40-fold excess of unlabeled HDL (**a**) and LDL (**b**), respectively, to record unspecific interactions. Specific association was calculated by subtracting unspecific values from total values. The results are represented as means±SEM of one triplicate experiment. EC50 for HDL and LDL were calculated with 103nM [95% confidence interval: 83nM-126nM] and 95nM [95% confidence interval: 79nM-125nM], respectively.

Supplementary Figure 5

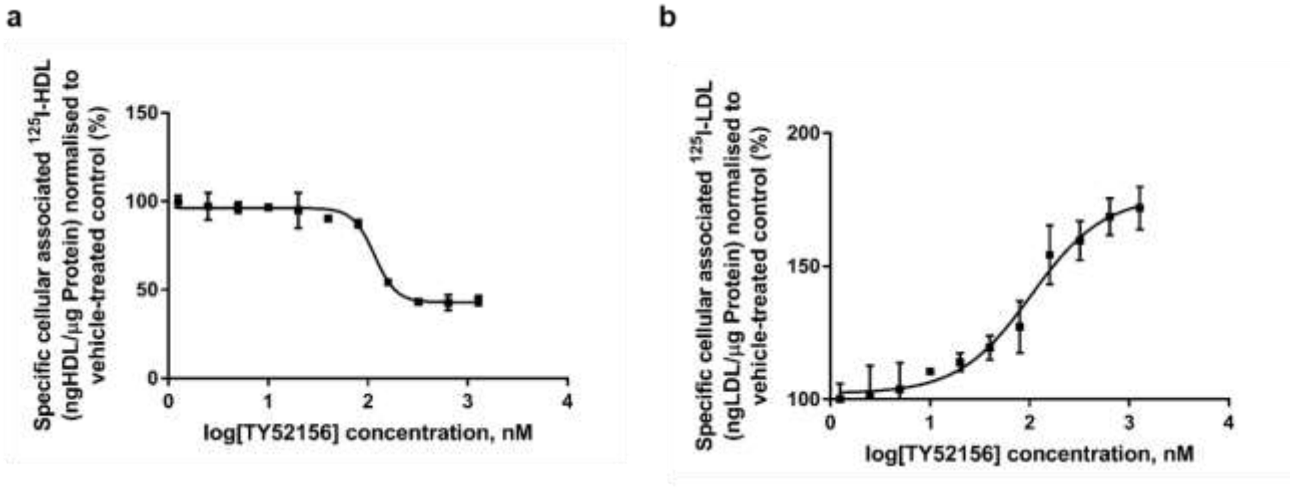

Log EC50 = 2.066 [116nM]
95% CI Log EC50 = 1.98-2.24 [98nM-173nM]

Log EC50 = 1.987 [104nM]
95% CI Log EC50 = 1.847-2.097 [70nM-208nM]

Supplementary figure 5: Dose-dependent effects of the S1P$_3$ inhibitor TY52156 on the specific association of ^{125}I-HDL (a) or ^{125}I-LDL with human aortic endothelial cells (HAEC). HAEC were cultured for 72hours and treated for 30 minutes with increasing concentrations of TY52156. HAECs were then incubated at 37 ºC for 1hour with 10µg/mL of ^{125}I-HDL (**a**) or ^{125}I-LDL (**b**) in the absence (total) or in the presence of 40-fold excess of unlabeled HDL (**a**) and LDL (**b**), respectively, to record unspecific interactions. Specific association was calculated by subtracting unspecific values from total values. The results are represented as means±SEM of one triplicate experiment. EC50 for HDL and LDL were calculated with 116nM [95% confidence interval: 98nM-173nM] and 104nM [95% confidence interval: 70nM-208nM], respectively.

Supplementary Figure 6

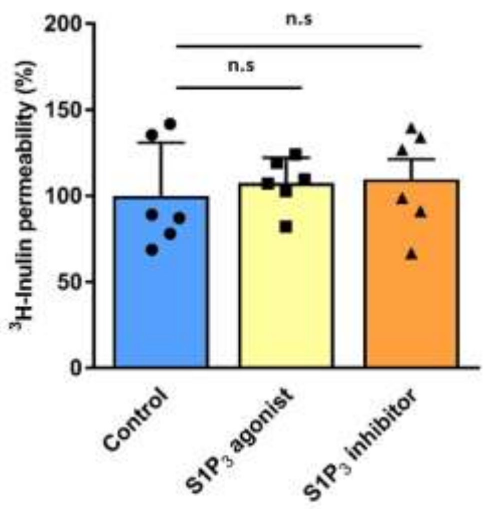

Supplementary Figure 6: Endothelial barrier function in the presence of S1P receptor inhibitors shows the filtration of tritiated inulin through HAECs cultivated in a transwell system: confluent cells were incubated for 30 minutes at 37 ºC with 100nM of S1P$_3$ agonist CYM5541 or 110nM of the S1P$_3$ inhibitor TY52156.

Supplementary Figure 7

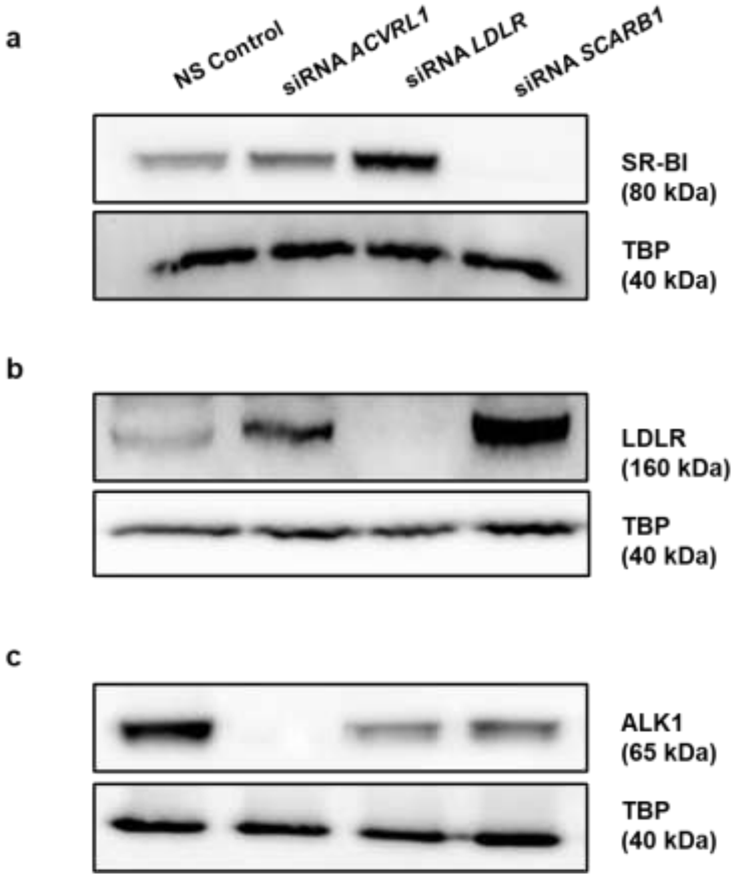

Supplementary Figure 7: Efficiency and co-regulatory effects of transfections with siRNA against *SCARB1, LDLR* and *ACVRL1* HAECs were transfected either with siRNA against *SCARB1* or *LDLR* or *ACVRL1* or with non-silencing siRNA (NS control) for 72 hours. Proteins of harvested and lysed cells were analyzed by western blots with antibodies against SR-BI **(a)** LDLR **(b)** or ALK1 **(c)** representative western blots and TATA-binding protein (TBP) used as the loading control.

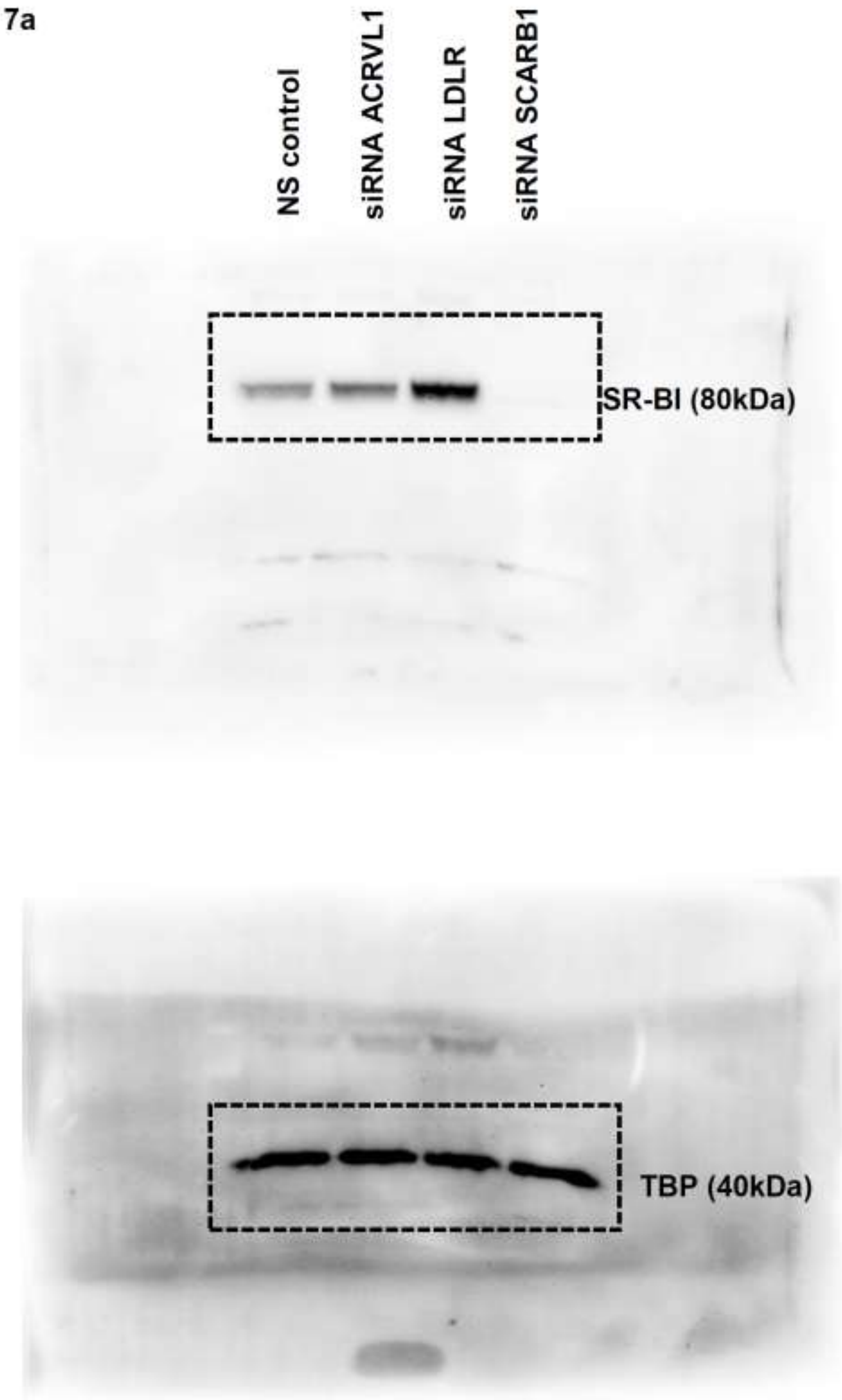

Supplementary Figure 7, continued: Complete and unedited gels of supplementary figure 7a

7b

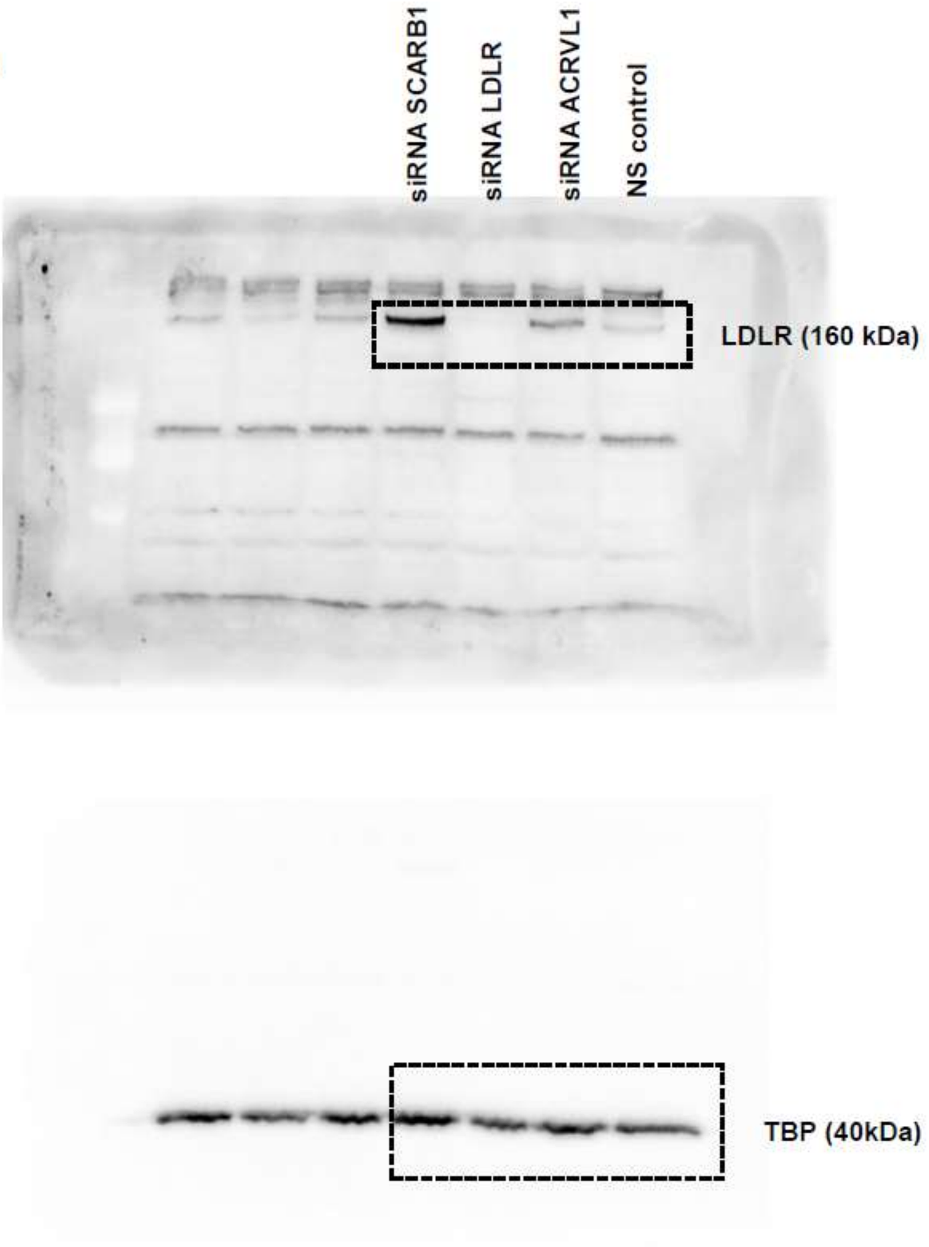

Supplementary Figure 7, continued: Complete and unedited gels of supplementary figure 7b

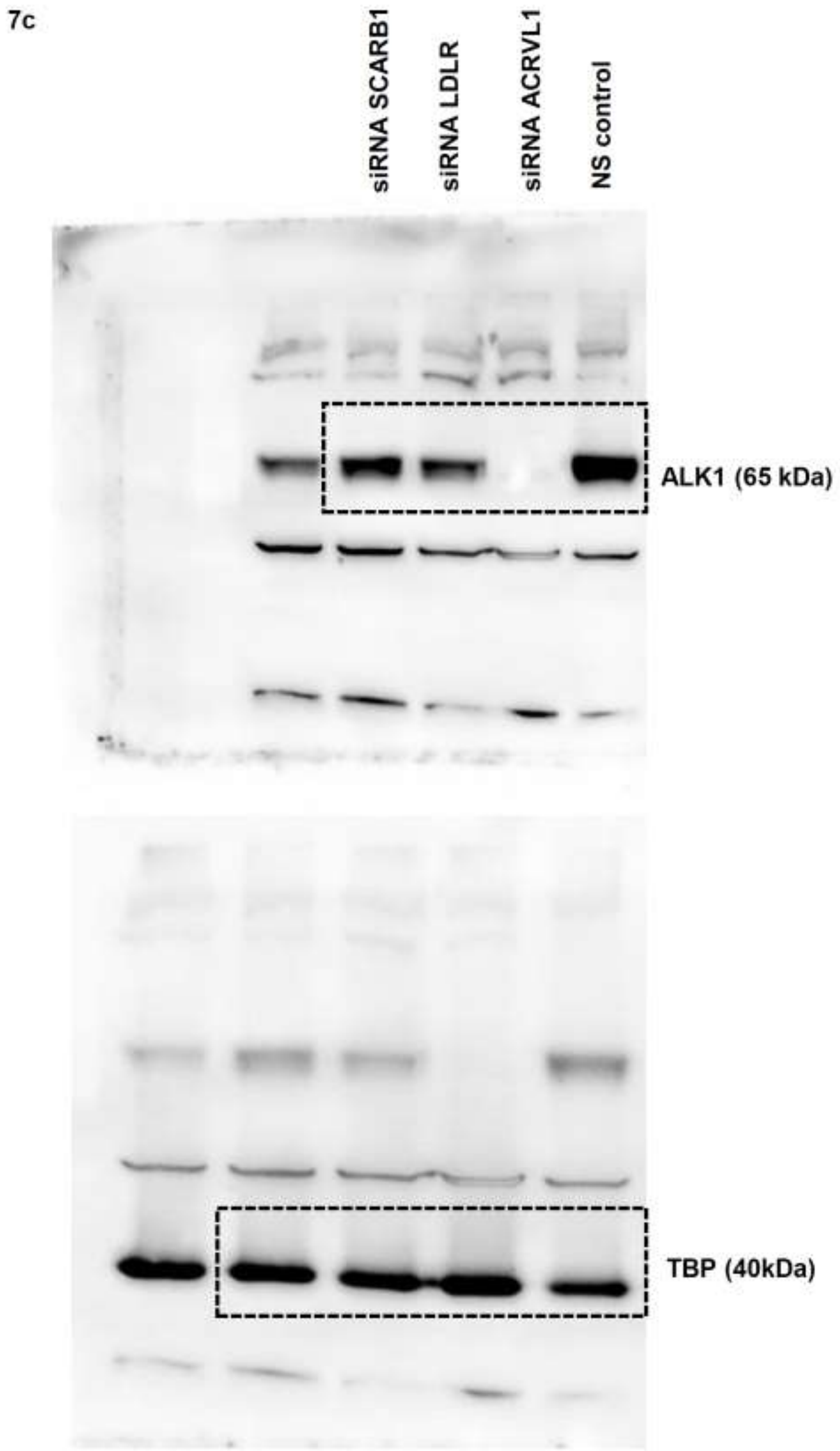

Supplementary Figure 7, continued: Complete and unedited gels of supplementary figure 7c

Supplementary Figure 8

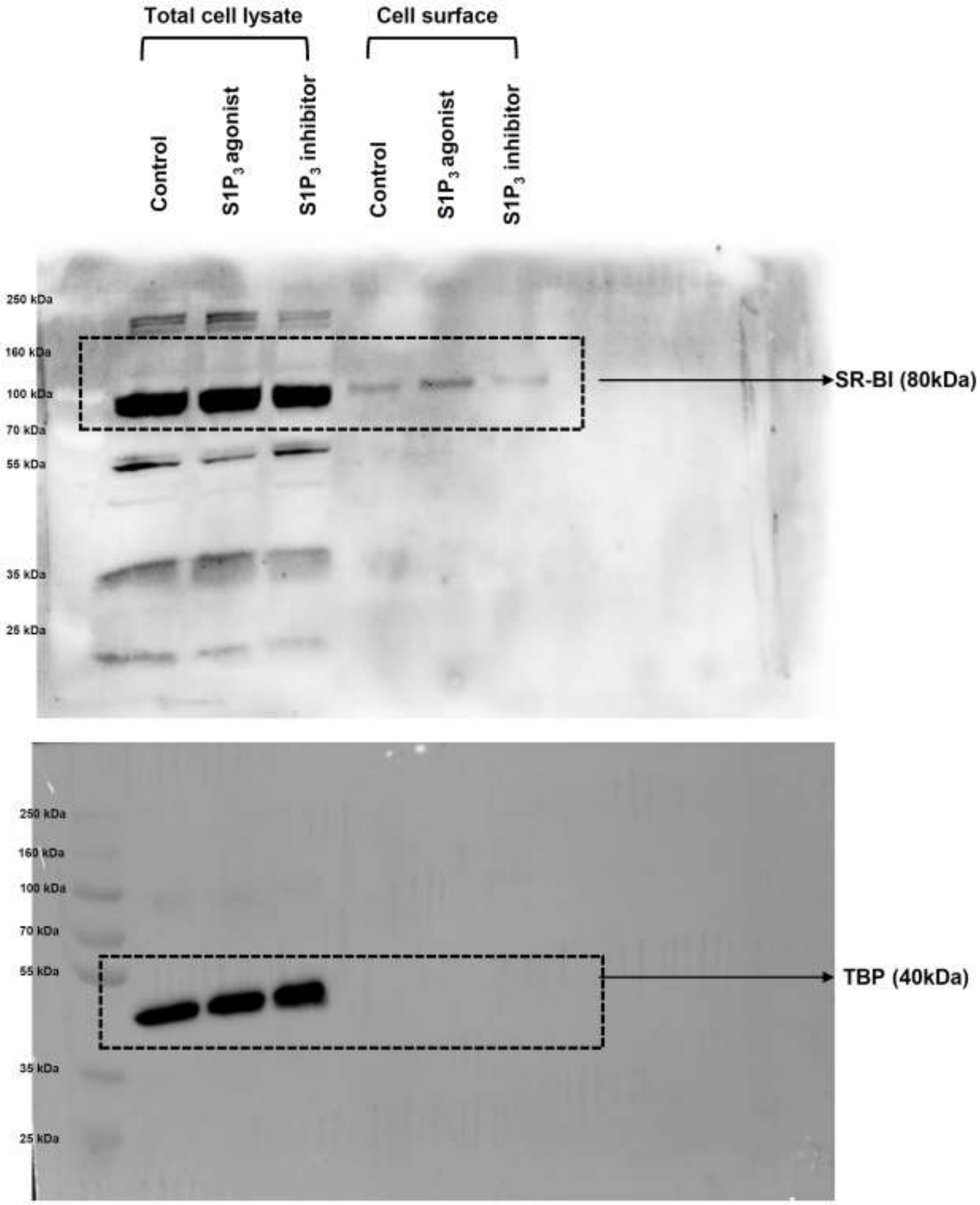

Supplementary Figure 8: Complete and unedited gels of figure 6a

Supplementary Figure 9

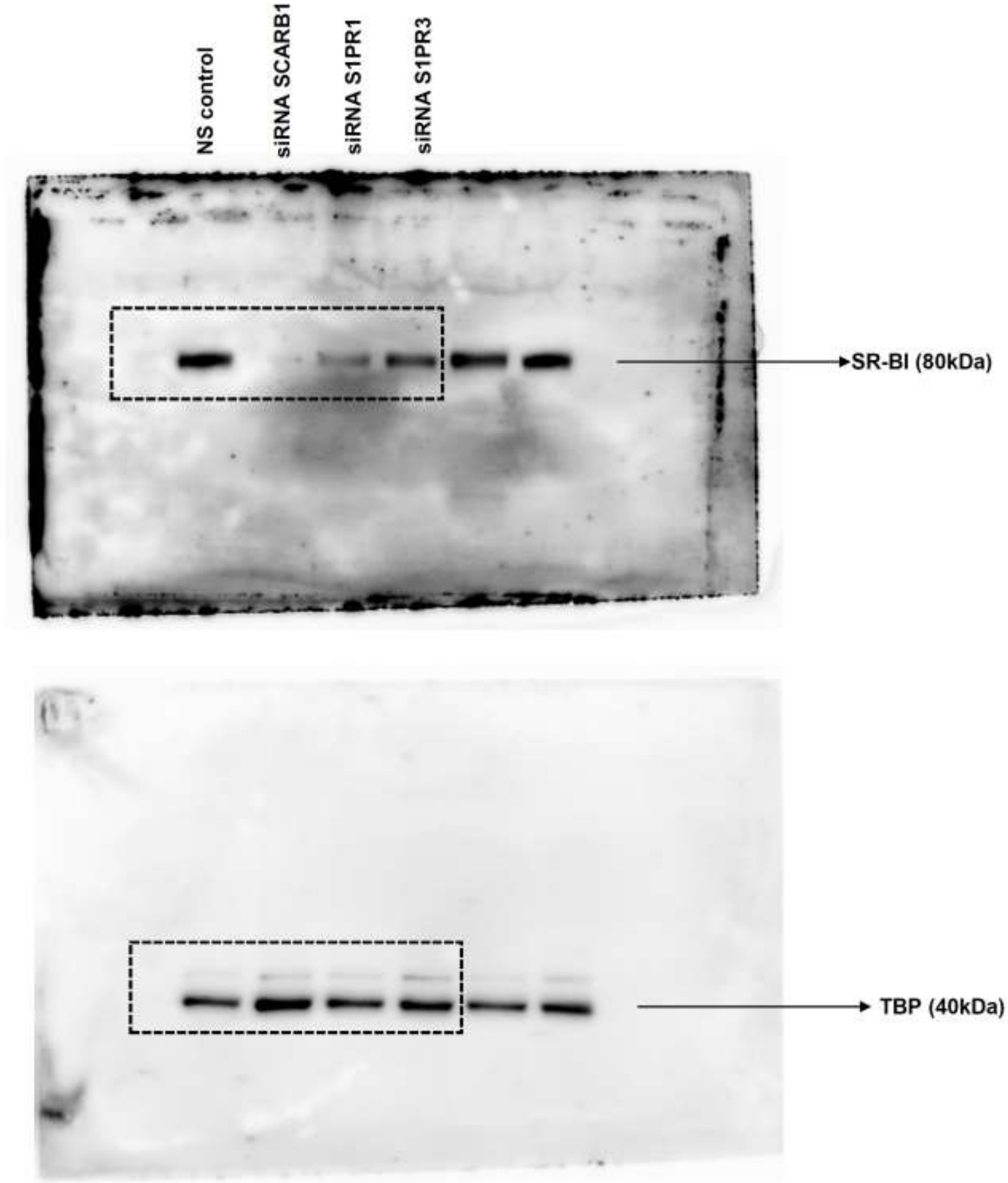

Supplementary Figure 9: Complete and unedited gels of figure 6d

Supplementary Figure 10

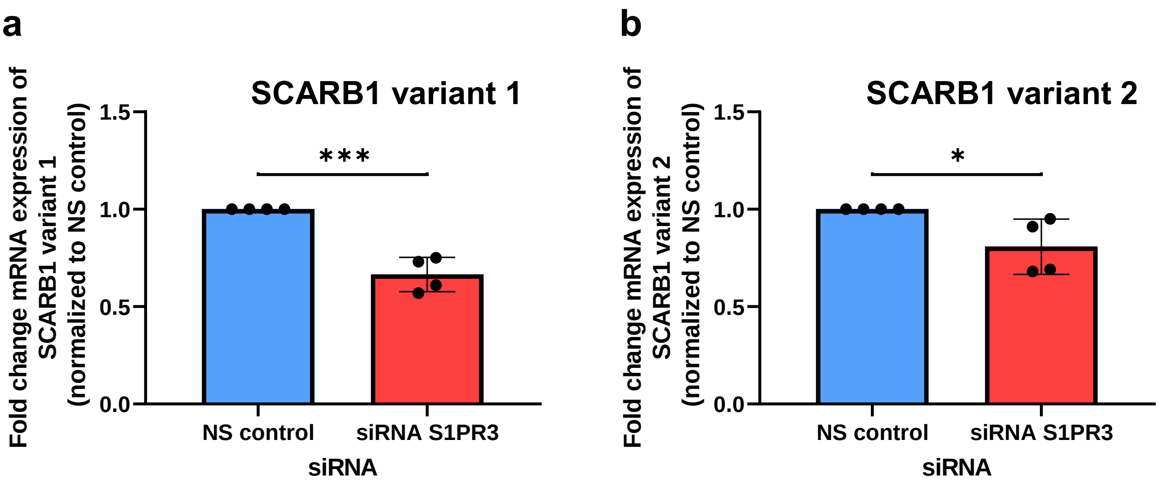

Supplementary Figure 10: RT-PCR of splice SCARB1 variants 1 and 2. The results are represented as mean±SD of four independent experiments. ***$P \leq 0.001$, *$P \leq 0.05$ (two-tailed Student's *t*-test).

Supplementary Figure 11

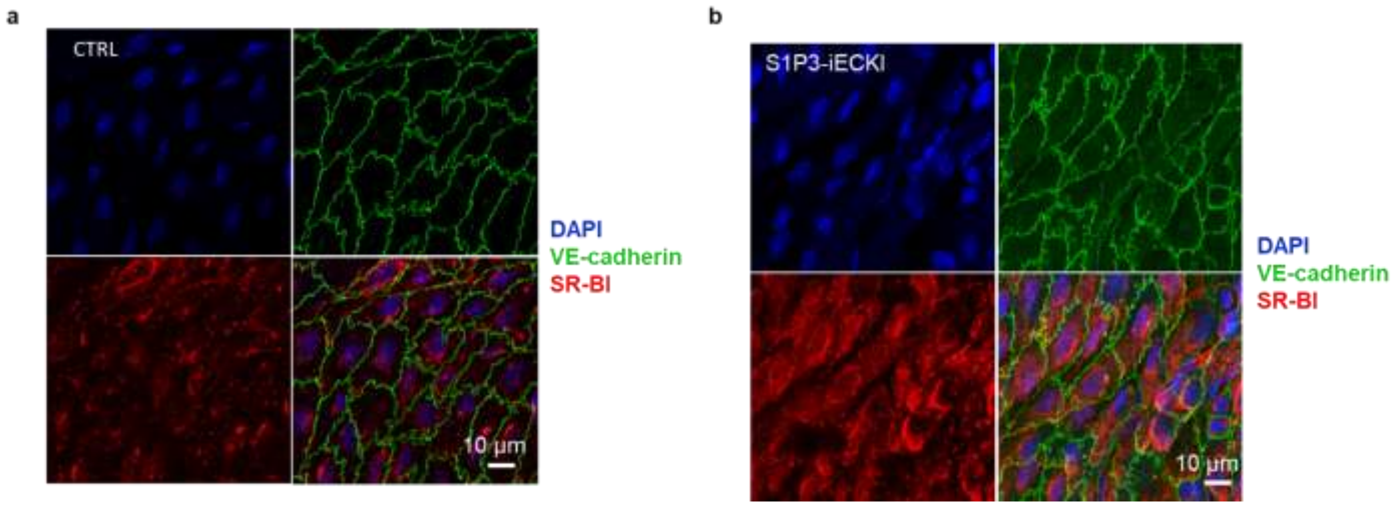

Supplementary Figure 11: Demonstration of SR-BI in the endothelium of aortas from *Apoe* haploinsufficient mice without (a; CTRL) or with overexpression of $S1P_3$ (b, S1P3-iECKI). Figure shows *en-face* prepared aortic immunostainings. Aortas were quickly cleared from the adventitial tissue, opened longitudinally, and incubated with primary and secondary antibodies conjugated with green or red fluorescent dyes, as indicated. Nuclei were counterstained with DAPI. Images were captured by confocal microscope and z-axis projections of 14 scanned planes are shown. Scale bar = 10µm.

Supplementary Figure 12

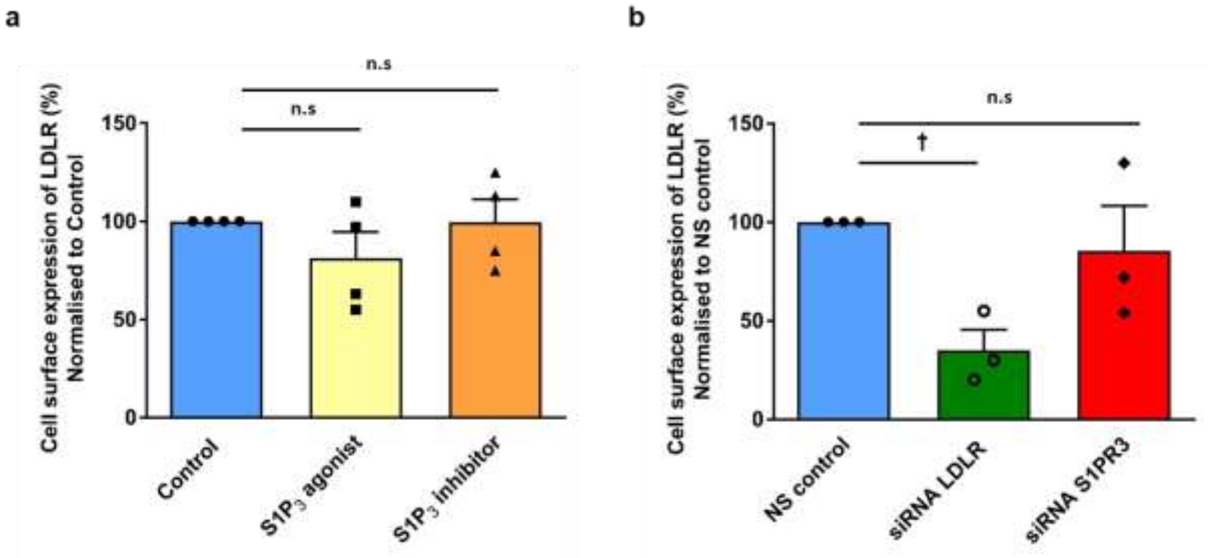

Supplementary Figure 12: Effects of drug (a) or RNA interference with S1P$_3$ (b) on LDLR cell surface abundance. LDLR cell surface levels were measured using **(a)** HAECs treated with either S1P$_3$ agonist (CYM5541, 100 nM) or S1P$_3$ inhibitor (TY52156, 110 nM) for 30 minutes at 37ºC, or **(b)** HAECs 72 hours post transfection with the indicated siRNAs. The results are represented as means±SEM and each data point represents the median fluorescent intensity in one of four **(a)** and in one of three **(b)** identical experiments, respectively. +P ≤ 0.05, n.s represents "not significant" (one-way ANOVA followed by Student-Newman-Keuls post hoc test).

Supplementary Figure 13

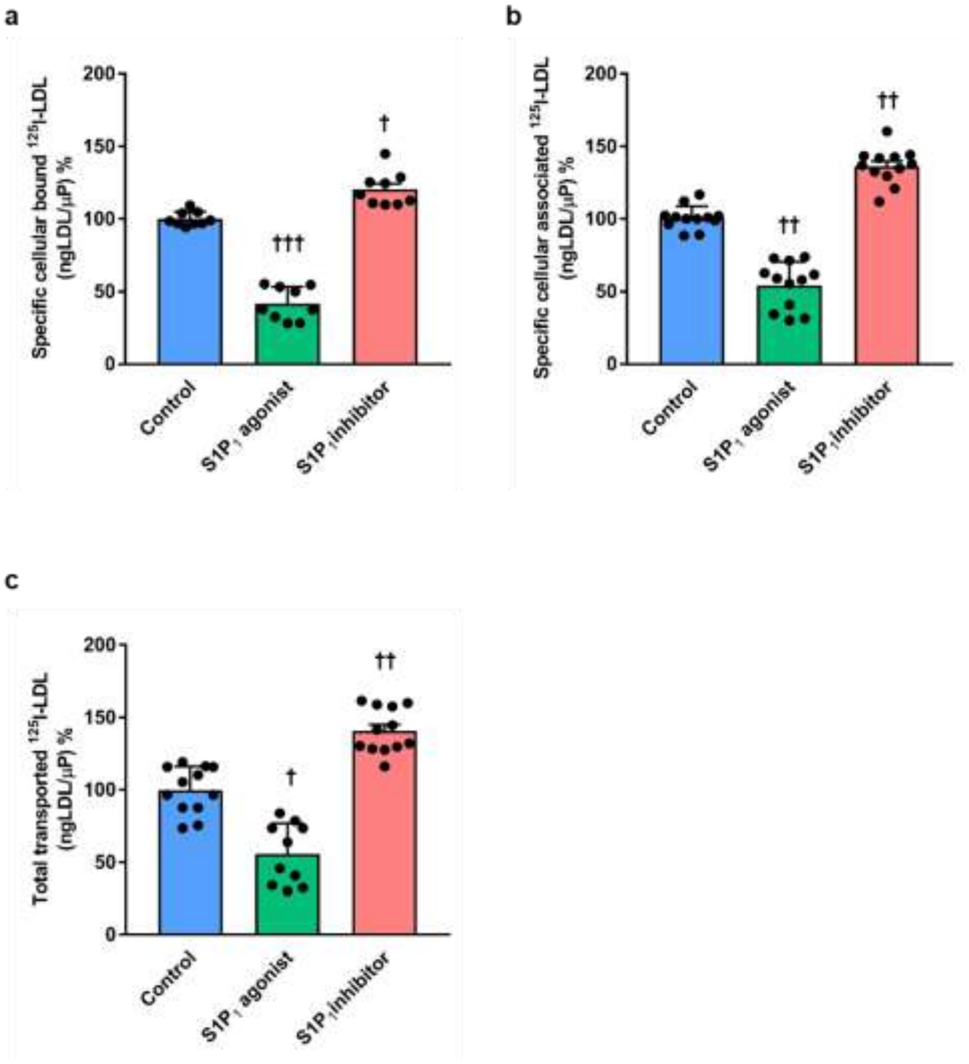

Supplementary figure 13: Both activation and inhibition of $S1P_1$ exert opposite effects on transendothelial binding, association, transport of LDL in HAECs HAECs were cultured for 72hours. Cells were then treated with S1P1 agonist (SEW2871, 20nM) or S1P1 inhibitor (W146, 20nM) for 30 minutes at 37 ºC. To study cellular binding (**a**) and association (**b**), HAECs were incubated with 10µg/mL ^{125}I-LDL for 1hour at 4ºC and 37ºC, respectively, in the absence (total) or in the presence of 40-fold excess of unlabeled HDL and LDL, respectively, to record unspecific interactions. Specific association and binding were calculated by subtracting unspecific values from total values. For the measurement of transport, HAECs were cultured on inserts. The transport of 10µg/mL ^{125}I-LDL (**c**) from the apical to basolateral compartment was measured after 1 hour incubation at 37 ºC. The results are represented as means±SEM of four independent triplicate experiments. +++$P \leq 0.001$, ++$P \leq 0.01$, +$P \leq 0.05$, n.s represents “not significant” (two-tailed Student’s *t*-test).
